# Integrative architecture and structural boundaries of the subdigital adhesive toe pads of *Gekko gecko* (Linnaeus, 1758) and guidelines for toe pad demarcation in other geckos

**DOI:** 10.1111/joa.70251

**Published:** 2026-09-27

**Authors:** Anthony P. Russell, Sonia M. Delannoy, Austin M. Garner

**Affiliations:** ^1^ Department of Biological Sciences University of Calgary Calgary Alberta Canada; ^2^ Department of Biological Sciences Florida Atlantic University Boca Raton Florida USA

**Keywords:** area, lamellae, phalanges, scales, scansors, setae, tendons

## Abstract

Many species of gecko and all but one species of anole possess enlarged, serially arranged scales on the ventral surfaces of their digits that collectively are referred to as toe pads. These are commonly considered key innovations and have been the subject of many studies within and beyond the biological sciences. The definition of squamate toe pads, however, remains vague. This is particularly problematic for comparative morphological and biomechanical studies wherein toe pad area is commonly employed as a dependent or independent variable or covariate. Lack of a standardized means of delimitation of toe pads impedes accurate measurement of these ecologically and evolutionarily important features and compromises effective comparison between species and studies. Herein, we take a comprehensive approach to the delimitation of the toe pads of the Tokay gecko (*Gekko gecko*), the species of gecko most frequently studied in relation to the phenomenon of gecko adhesion. In addition to consideration of the broadly adopted, but somewhat arbitrarily delineated, circumscription of toe pads based on characteristics of scale dimensions alone, we also investigate their boundaries by examining the relationship between subdigital scales and subcutaneous anatomical features (employing osteological and tendon‐based [TB] criteria). We find that the methods employing only external dimensions of subdigital scales to delimit toe pads overestimate their area, thereby resulting in functional studies that likely underestimate toe pad adhesive stress (force per unit area). Of the two approaches that anatomically delimit toe pads, that based upon tendinous connections (demarcating what we refer to as the LDT region of the digits) encompasses all epidermal filaments that are true setae (those bearing spatulate tips), the only structures empirically validated to facilitate whole‐animal adhesion. As such, we encourage future studies to employ the TB approach to accurately delimit gekkotan (and anoline) toe pads in a functionally and anatomically meaningful (and standardized) fashion and provide guidelines about how this can be achieved.

## Graphical abstract

The adhesively competent toe pads of *Gekko gecko* (and other geckos) are delimited by a combination of external and deep anatomical features and cannot be identified based upon external scale form alone. We encourage future investigators to delimit toe pads in this functionally and anatomically meaningful fashion.

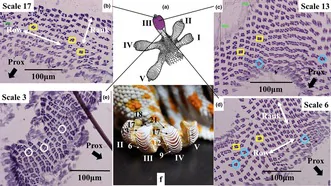

## 1 INTRODUCTION

The subdigital surfaces of many geckos and all but one anole (Peterson & Williams, 1981) bear a series of enlarged, modified scales that collectively constitute what are referred to as toe pads. All the enlarged scales incorporated into visually identified toe pads are tacitly assumed to bear setae (Garner & Russell, 2021) on their exposed plantar surface and to participate in adhesive interactions with the locomotor substratum. Features of the toe pads of lizards (such as the form and location of the included scales, the presence or absence of claws, and internal anatomical attributes—see Russell & Gamble, 2019) have been examined in a variety of studies, although statements specifying what exactly delimits them remain rather vague. Unlike the appendicular attachment organs of hylid frogs (Barnes et al., 2006; Green, 1981), disc‐winged bats (Riskin & Fenton, 2001; Wimsatt & Villa‐R, 1970), mouse lemurs and tarsiers (Nachtigall, 1974), feathertail gliders (Rosenberg & Rose, 1999) and hexapod arthropods (Federle, 2006; Gorb & Beutel, 2001), those of geckos are not discretely demarcated from adjacent regions of the integument. Instead, the digits of pad‐bearing geckos often exhibit a gradation from the attachment structures to juxtaposing scales. The absence of an objectively based definition is, therefore, surprising because the toe pads of geckos and anoles are considered to be key evolutionary innovations (e.g., Burress & Muñoz, 2022; Garcia‐Porta & Ord, 2013; Losos, 2009; Miller & Stroud, 2022; Nicholson et al., 2006; Peterson, 1983). Toe pads have arisen independently within the Gekkota on numerous occasions (Gamble et al., 2012; Russell & Gamble, 2019) and their morphological diversity is extensive, with at least 15 distinctive morphological configurations (Garner & Russell, 2026; Russell & Gamble, 2019, figure 2). There is also increasing evidence of the existence of incipient stages of toe pad expression within various gekkotan lineages (Ginal et al., 2026; Riedel et al., 2021, 2024; Russell et al., 2015) that endow such species with whole‐animal adhesive competence (Ginal et al., 2026; Higham et al., 2017). Thus, some means of objectively defining the boundaries of these evolutionarily and ecologically important features (Garcia‐Porta & Ord, 2013; Irschick et al., 2006) is essential because functional attributes of the subdigital adhesive system are routinely extrapolated from calculations based upon toe pad area (Cobos & Higham, 2022; Griffing, Daza, et al., 2024; Griffing, Gamble, et al., 2024; Howell et al., 2022; Michaud et al., 2023; Pillai et al., 2020a; Wright et al., 2021).

Since measurement of toe pad area has never been standardized by the application of any objective criteria, it is likely that at least some of the variability reported for area‐associated assessments of adhesive performance (Autumn et al., 2000; Autumn & Peattie, 2002; Bauer & Good, 1986; Higham et al., 2017; Irschick et al., 1996) stems from the application of different criteria to establish toe pad boundaries. In such investigations, no morphological attributes, beyond the incorporation of a selected set of scales, have been employed to define toe pads. Furthermore, the setae that are carried on the modified toe pad scales have been assumed to be of uniform presence, distribution, and structure (Autumn, 2016) across the entirety of the delineated toe pad (despite earlier investigations of setae indicating intraspecific variability of some dimensional attributes—Bauer & Good, 1986; Haase, 1900; Maderson, 1970; Ruibal & Ernst, 1965), regardless of the criteria employed to circumscribe it. Such assumptions lead to the corollary that each individual enlarged, modified scale (referred to in the literature as lamellae and/or scansors) included within the toe pad (however delimited) will contribute to the total adhesive potential of the toe pad in direct proportion to its area.

The potential importance of subdigital pad area as an indicator of adhesive ability, and the lack of application of defining features for the subdigital pad, prompted us to undertake a more holistic examination of what constitutes a toe pad. This is especially important for comparative investigations because, as indicated above, toe pad configuration varies between lineages (Gamble et al., 2012; Garner & Russell, 2026; Russell, 1976; Russell & Gamble, 2019). In comparative studies of function, it is important that functionally equivalent (i.e., analogous) entities are assessed. Variation in the structure of the subdigital scales and the epidermal filaments they carry requires in‐depth investigation to enable determination of the following questions. How, if at all, do the scales and their filamentous outgrowths (Garner & Russell, 2021) vary along the length of the digits? Do all scales along the length of the digit carry adhesive setae and are these structures homogeneous in form within and/or between scales, digits, and limbs? Is spacing between setae and the density of setae consistent along the length of the digit? How, if at all, does the geometry of filamentous outgrowths relate to their position along the digit? Can the form of the filaments be related to function?

Although over 2400 species of limbed gekkotans have been described (Uetz et al., 2026), approximately 60% of which are regarded as possessing adhesive toe pads, only one species, *Gekko gecko* (the Tokay gecko), as noted by Bauer (2019), has been intensively investigated in relation to the adhesive properties of its setae (Autumn et al., 2000, 2002; Bauer & Good, 1986; Garner & Russell, 2026; Gennaro, 1969; Hill et al., 2011; Huber et al., 2007; Huber, Gorb, et al., 2005; Huber, Mantz, et al., 2005; Irschick et al., 1996; Maderson, 1964, 1966, 1970; Ruibal & Ernst, 1965; Russell, 1975, 1979, 1981a, 1981b, 1986; Stewart & Daniel, 1972; Stork, 1983; Sun et al., 2005; Xu et al., 2015, 2018). We thus use this species to explore digital structure and the range of expression of filamentous elaborations carried thereon.

The subdigital pads of *Gekko gecko* (Figure 1a,b) are evident externally as a series of transversely enlarged, modified scales (Russell, 1975). Although such scales are generally treated as being homogeneous in structure, Russell (1981a, 1986) divided them into two subsets: scansors, which are the distal enlarged plates associated with the hyperextensible portion of the digit (posited to be most directly involved in adhesion), and a series of more basally situated lamellae, which are anatomically less complex (Bergmann & Russell, 2003; Johnson & Russell, 2009; Russell, 1981a, 1986, 2002; Russell & Oetelaar, 2016; Webster et al., 2009).

**FIGURE 1:**
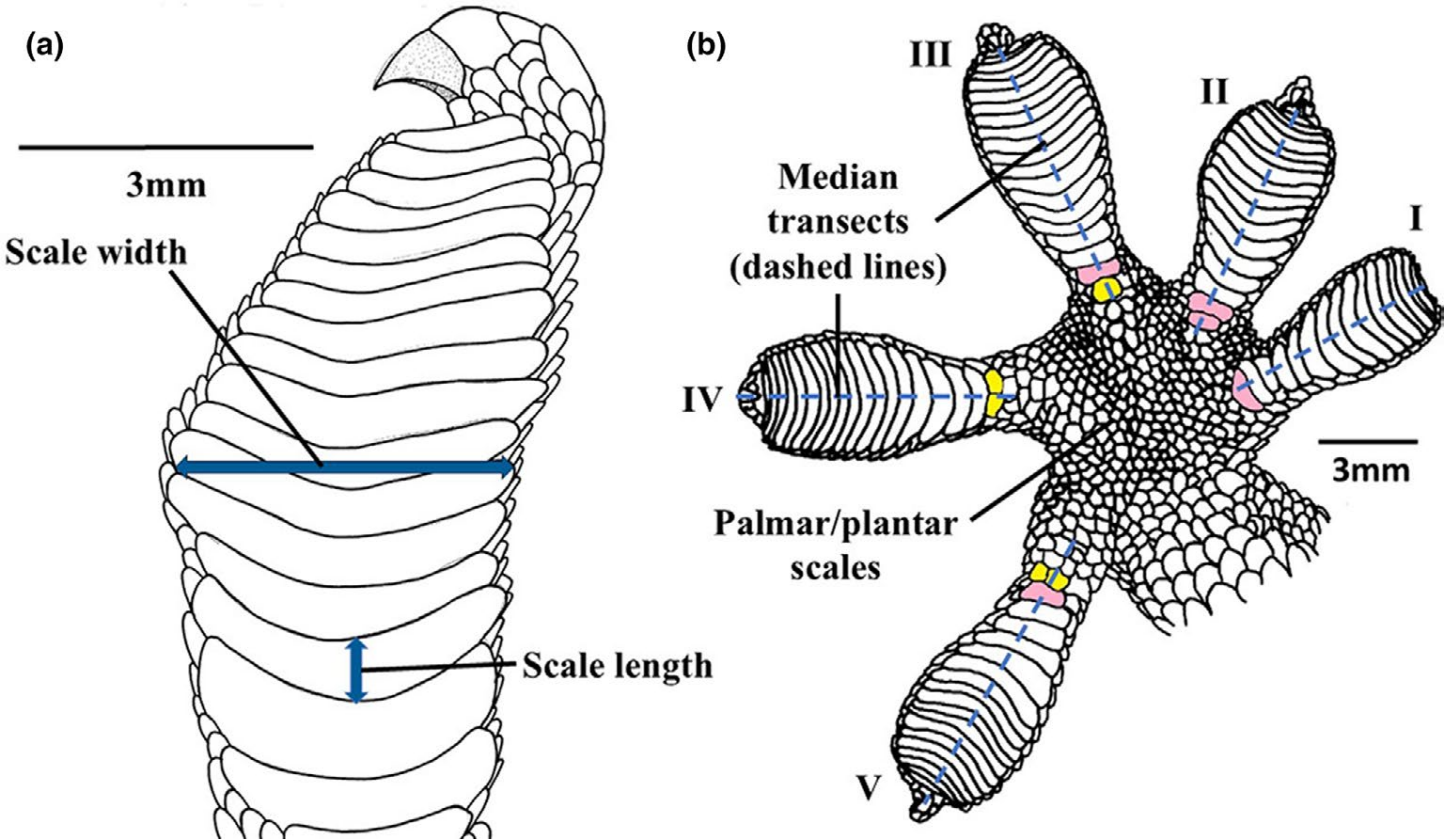
Ventral aspect of the digits of *Gekko gecko*. (a) digit IV, left pes indicating how the measurements for scale width and length were taken. (b) Ventral view of the left pes (drawn from a photograph of an individual adhering to a vertical pane of glass). Digit I is the shortest digit and lacks a claw. The median transect lines for observing the filaments on the subdigital surfaces are indicated. The proximalmost subdigital scales with a width‐to‐length ratio >1 are indicated in pink, and those wider than 2.5× the average palmar/plantar scale width are indicated in yellow (see text for details).

Russell et al. (2007) briefly considered some aspects of intraspecific setal field structure and variation in *Gekko gecko*, reporting on data for digit IV of the right manus of a single individual. They documented the length and surface area of the individual subdigital scales, the average number of filament rows on each subdigital scale, filament length, basal diameter, tip width, tip area, and tip density for each examined scale. They considered these parameters in relation to the mechanical operation of the setal fields during adhesive attachment and release. We here employ similar methodology to explore what definitively constitutes the toe pads on all digits of this species, and how toe pad area is apportioned between individual digits, as well as between the forelimbs and hindlimbs.

To more effectively define toe pads in terms of the scales that are mechanically able to be controlled by the hierarchical anatomical apparatus responsible for effecting adhesive contact and release (Russell, 2002), we take a stratified approach. First, we focus on external characteristics of the subdigital scales to determine their position relative to deeper anatomical attributes of the digits. Scale length, width, and area are examined to determine how external morphological attributes are integrated with underlying internal anatomy. We then examine subdigital structure via scanning electron and light microscopy to assess the correlation between the gross anatomy of the digits and the fine structure of the epidermal filaments that are responsible for their adhesive properties. This integrative approach allows us to identify potential differences among, and between, manual and pedal digits, and differences associated with previously employed criteria for the circumscription of the toe pads of *Gekko gecko*. Additionally, we consider our findings in the context of digital anatomy across the Gekkota to suggest a means of identifying the proximal boundary of toe pads in general.

## 2 MATERIALS AND METHODS

### 2.1 Specimens

Twenty‐five Tokay geckos (*Gekko gecko*) were acquired from an animal supply company and housed at the University of Calgary Animal Care Facility in accord with animal care protocol BI2002‐013. Each individual was identified by number and housed in its own terrarium in an animal care room at 25°C, with a photoperiod of 12 h light/12 dark, fed calcium‐dusted mealworms daily, and crickets every fifth day and provided water ad libitum. Every twelfth day, each gecko was weighed (using a 100 g Pesola balance) and measured to record snout‐vent length (SVL). Following measurement, each animal was placed in a clean terrarium.

To avoid the accumulation of debris and the effects of wear of the epidermis (Irish et al., 1988; Pillai et al., 2023) from influencing our results, geckos were euthanized by intraoral application of T61 (Hoechst Pharmaceuticals) 5–7 days after sloughing, thereby standardizing the sampling of the integument at the “perfect resting stage” (Maderson et al., 1970). Specimens were set prone, in a splayed posture, with digits separated (Figure 2), fixed in 10% neutral‐buffered formalin in a sealable setting tray for 1–2 days, rinsed in running tap water for 8–12 h, blotted dry, and stored supine in sealable trays in 70% ethanol.

**FIGURE 2:**
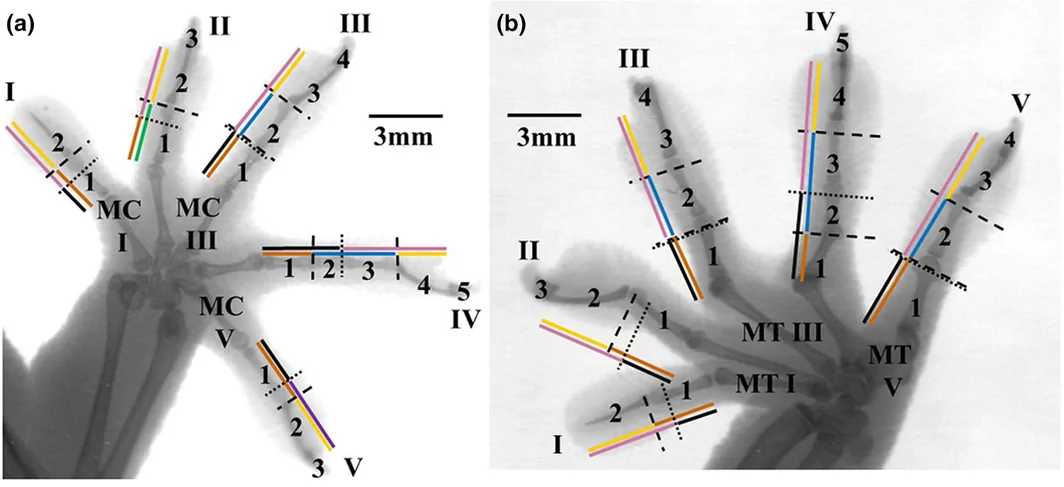
Radiographic dorsal view of the right manus (a) and right pes (b) of *Gekko gecko* (specimen 03 included in this study). Digits are identified by Roman numerals I–V and the phalanges of each digit are identified by Arabic numerals 1–5, with the basal phalanx of each digit bearing the number 1 (additional phalanges are identified by numbers in increasing sequence from proximal to distal). The outlines of the soft anatomical extent of the digits are evident. For the osteologically based (OB) subdivision of the digits (see the text for details), the extent of the proximal, intermediate and distal zones (identified by brown, blue, and yellow lines, respectively) are indicated for each digit; the boundaries between these zones are marked by dashed lines. For the tendon‐based (TB) subdivision of the digits (see the text for details), the basal and lateral digital tendon (LDT) zones (identified by black and lilac lines, respectively) are indicated for each digit; the boundaries between these zones are marked by dotted lines. Metacarpals (MC) and metatarsals (MT) I, III, and V are indicated.

### 2.2 Digit gross anatomy

Radiographs (Figure 2) of the manūs and pedes of each specimen were taken on Polaroid® Type 55 black and white positive/negative film, using a Hewlett‐Packard Faxitron model 43805N radiology unit, and scanned as digital images for measurement. Phalangeal lengths of the manus and pes were measured using ImageJ version 1.30 (National Institutes of Health, USA). These lengths, along with the digital margins clearly visible on the radiographs (Figure 2), allowed us to determine which subdigital scales were associated with which phalanx.

Three male and three female adult specimens (Table 1) were selected for comprehensive documentation of their digits. Images of the subdigital and palmar (manual)/plantar (pedal) surfaces of the right and left manus and pes were taken with a Nikon Coolpix 990 camera. Scale length (Figure 1a) was measured along a median transect line for each digit (Figure 1b) and scale width was measured across the width of the digit from its medial to its lateral border (Figure 1a), using ImageJ version 1.30 (National Institutes of Health, USA). Palmar and plantar scales (Figure 1b) were measured using ImageJ version 1.30 to determine the average width of these for each specimen.

**TABLE 1:** Measurements for *Gekko gecko* specimens used for this study (*n* = 6).

Identification number	Sex	Snout‐vent length (mm)	Weight (g)
02	F	141.3	40.5
03	M	146.8	47.5
06	M	148.6	70.5
07	M	153.0	57.5
11	F	139.7	44.0
16	F	145.4	56.5

*Note*: Specimen numbers relate to the complete sample of 25 individuals from which this subset was selected (see text for details).

Based upon previous purported assessments of toe pad area, we employed four approaches (see below) for obtaining this metric, permitting us to compare the outcomes. We incorporated various combinations of scales into the putative toe pad, in accord with these approaches.

The criterion advocated by Peterson and Williams (1981) for anoles, based upon externally visible features, includes all subdigital scales with a length‐to‐width ratio greater than one (Figure 1b) in the measurement of toe pad area. Similarly, the criterion advocated by Bauer and Good (1986) for *Gekko gecko* includes all scales with a width 2.5 times or more than that of the average palmar scale in the calculation of toe pad area (Figure 1b). Other authors employing toe pad dimensions in their studies have either provided no specification about the proximal boundaries of the toe pads beyond a subjective assessment of scale dimensions, or merely simply state that the toe pad area was measured without indicating any bounding conditions (e.g., Crandell et al., 2014; Gilman et al., 2015; Howell et al., 2022; Irschick et al., 1996, 2005; Labonte et al., 2016; Macrini et al., 2003; Niewiarowski et al., 2008; Stuart et al., 2014).

Two anatomically based criteria (employing internal structural markers) have also been employed to assign scales to the putative toe pad (Figure 2). The first (hereafter referred to as the osteologically based [OB] criterion) employs phalanges (Figure 2) as indicators of the proximal extent of the toe pad (Rothier et al., 2022; Russell & Garner, 2021). Rothier et al. (2022) examined only manual digits III, IV, and V of geckos. They regarded the proximal end of the penultimate phalanx (Figure 2) as demarcating the proximal limit of the hyperextensible portion (toe pad) of the digits and assigned more proximal phalanges to intermediate (their “medial”) and proximal regions. They did so without reference to digits I and II. Our assignment to OB positional categories (see below) differs from that of Rothier et al. (2022) to ensure consistency of application across all digits. Thus, our distal region is identical to that reported by Rothier et al. (2022), but assignment to basal and intermediate regions differs for some digits. Radiographic images were used to determine to which of three topographical regions (proximal, intermediate, and distal) each subdigital scale is assignable (Figure 2). We define these regions anatomically as follows: the proximal region encompasses all subdigital scales lying beneath the basal phalanx (phalanx 1) of each digit (Figure 2); the distal region encompasses all subdigital scales lying beneath the penultimate phalanx of digits II–V and the elongate distalmost phalanx of digit I (manus and pes; digit I being clawless) (Figures 1b and 2); the intermediate region encompasses all subdigital scales lying beneath any phalanges that intervene between the proximalmost phalanx and the penultimate phalanx of digits III and IV of the manus and III, IV, and V of the pes (Figure 2). In comparison with the assignments of Rothier et al. (2022), our intermediate zone of manual digit IV includes two phalanges and the basal region just one (rather than vice versa).

The second anatomically based criterion (hereafter referred to as the tendon‐based [TB] criterion) recognizes only two regions (applicable equally to all digits), a distal zone (Figure 2) comprising scales integrated with the lateral digital tendons (Figure 3d) (termed the LDTs region) and a basal region (Figure 2). The distinction between these regions is dependent upon the degree of association of the subdigital integument with deeper tissues (Russell, 1986). The skin of the digits is removable without impediment from all regions dorsally and from their bases ventrally (Figure 3e,f). The ventral integument in the LDT region of each digit is firmly bound to the LDT system (Figure 3d–f) and cannot be removed when the autopodium is skinned. This zone coincides with the region of the digit encompassed by the penultimate phalanx (and its equivalent in digit I) and a portion or the entirety of the adjacent antepenultimate phalanx, and even the second phalanx of digit IV (Figure 3b), the two latter locations belonging to the OB intermediate region (see above) of those digits possessing such a zone (Figure 2). The TB subdivision also coincides with the extent of the digital vascular sinuses (Figure 3c) and their associated reticular networks (Russell, 1975, 1981b) that occupy the bases of the imbricating, enlarged subdigital scales in this region (Figure 3c).

**FIGURE 3:**
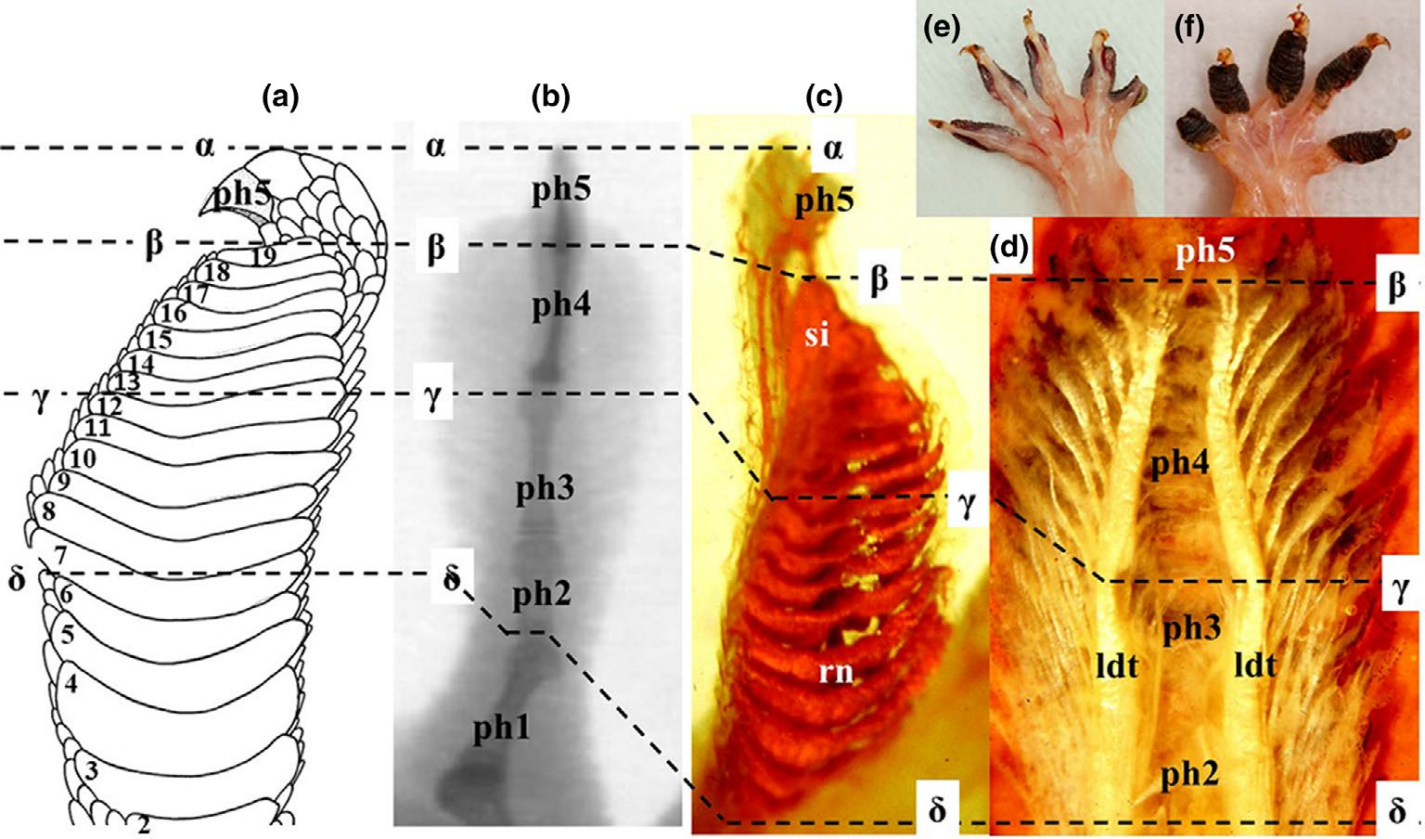
Anatomical features of the digits of *Gekko gecko*. (a) Ventral view of digit IV, left pes (as seen in Figure 1a) with subdigital scales numbered in reverse order from proximal to distal (see text for details). (b) Radiograph of digit IV, left pes in dorsal view (as seen in Figure 2b) with the phalanges (abbreviated ph) numbered sequentially from proximal to distal. (c) Digit IV, left pes with the digital sinus and reticular vascular network system injected with Microfil® and the soft tissues cleared of pigmentation. (d) Digit IV, left pes dissected from the dorsal aspect with the phalanges removed and the lateral digital tendon system displayed. (e) Dorsal view of the left manus of a freshly euthanized specimen with the integument stripped away, showing the complete removal of the skin from this aspect and the clear outlines of the toe pads remaining intact. (f) Ventral view of the left manus of the same specimen as in panel e with the integument stripped away, showing the toe pads of all five digits remaining in place and undamaged. Ldt, lateral digital tendon; ph, phalanges (or their location when removed), indicated by Arabic numbers; rn, reticular network within the bases of subdigital scales; si, vascular venous sinus located ventral to ph 4. Horizontal dashed lines link equivalent points on the images, as follows: Line *α* (alpha) links the distal end of ph 5 (the claw); Line *β* (beta) links the location of the distal extremity of the distalmost enlarged subdigital scale. Line *γ* (gamma) links the location of the articulation between phalanges 3 and 4; Line *δ* (delta) links the location of the shaft of phalanx 3 immediately proximal to its expansion into its widened distal articulatory process and the proximalmost point of branching of the lateral digital tendons. Approximate scale can be gleaned from Figures 1a and 2a—all images are from different individuals.

Areas of the subdigital regions pertinent to each of the four approaches outlined above were determined using ImageJ version 1.30 (National Institutes of Health, USA). Statistical comparisons of regional areas (for those approaches that subdivide the digits in this way) were conducted for all digits by application of a repeated measures mixed procedure analysis in SAS version 8 (SAS Institute Inc.). Separate analyses were conducted for areas of the OB demarcated regions (Figure 2: proximal, intermediate and distal) and the TB demarcated regions (Figure 2: basal and LDT). Sources of significance were identified with post hoc pairwise comparisons and Dunn‐Sidàk adjusted *α* levels. The following comparisons were made: (1) area of the regions occurring within individual digits; (2) area of each identified region on all digits of the manus; (3) area of each identified region on all digits of the pes; and (4) area of each designated region on serially homologous (Bryant et al., 1996) digits (e.g., digit I of the manus and digit I of the pes).

### 2.3 Digit microscopic anatomy

#### 2.3.1 Scanning electron microscopy

Features of the filaments (setae, prongs, spines, and spinules; see Garner & Russell, 2021) carried on the subdigital and other scales were ascertained via scanning electron microscopy and light microscopy. Digits of six specimens (Table 1) were employed for investigation of the Oberhaütchen (Maderson, 1966, 1970) elaborations carried on their subdigital scales. All digits of the left and right manus and pes were examined using environmental scanning electron microscopy (ESEM). Median longitudinal sections (Figures 1b and 4) of the digits were examined.

**FIGURE 4:**
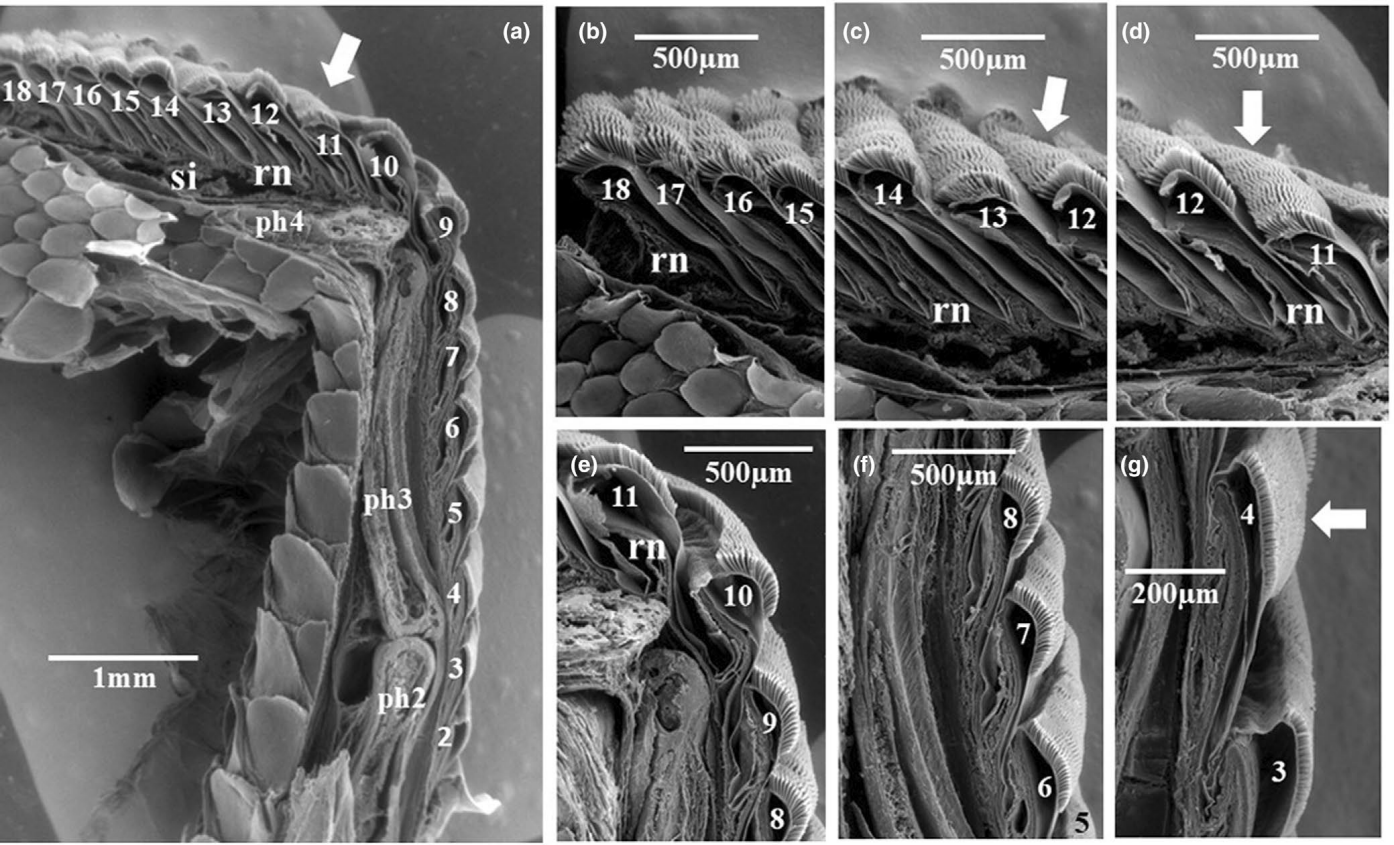
Scanning electron microscopic images of the median transect (see Figure 1b) of digit IV, right pes of *Gekko gecko* (specimen 11, see Table 1). A. The entire digit, with subdigital scales numbered in reverse order from proximal to distal (see Figure 3a for a depiction of this convention). Panels b–g show higher magnifications of sets of enlarged subdigital scales, with scale numbers corresponding to those shown in panel a. The broad white arrows in panels a, c, d, and g indicate the orientation from which views were obtained of the setal tips when the SEM stub was reoriented to enable orthogonal views of the subdigital surface. ph 2, ph 3, ph 4, phalanges 2, 3, and 4; rn, location of reticular vascular network system; si, location of digital sinus.

To facilitate comparison of digital features within and between individuals, the distalmost enlarged scale was employed as the common registration point (Figure 3a) and assigned the highest number in its own series, with the lowest number being situated at the base of the digit (Figures 3a and 4). Different digits have different lengths within and between individuals (Figures 1b and 2) and carry different numbers of subdigital scales (Figure 1b). The distalmost (and highest numbered) scale of each digit (Figures 3a and 4) is always incorporated in assessments of the toe pad of each digit, regardless of how the toe pad is “defined”, and thus serves as an operational marker of the distal extremity of the toe pad applicable to all approaches used for toe pad definition (see above).

All samples for SEM examination were dehydrated through a graded ethanol series prior to treatment in a Seevac CO_2_ critical point dryer. Dried samples were mounted with colloidal silver paste on Zeiss aluminum flat endpin SEM mounts and sputter coated with gold in a Hummer II Sputter Coater. Imaging employed a Philips/FEI ESEM XL30 in the University of Calgary Microscopy and Imaging Facility, Cumming School of Medicine. To minimize charging effects, all images were taken under variable pressure in the GSE (gaseous) mode of the microscope.

The aspect ratio of the images of longitudinally sectioned digits (Figure 4) was corrected using XL TIFF Stretch version 2.0 (© M.T. Otten) to assure that they had a 1:1 ratio before measurement of epidermal filaments was undertaken.

We measured filaments on all subdigital scales included in the OB distal and intermediate, and the TB LDT regions (Figure 2). Critical point drying resulted in the outer epidermal generation near the base of the digits of several specimens sloughing off (due to integumentary dissociation at the plane through which the digit was severed), rendering imaging of the basal subdigital scales more difficult. Measurements for these scales were only possible for a reduced number of specimens. Averages of measurements of epidermal filaments were calculated from the measurements recorded for all specimens. Lengths, basal diameters, and tip widths of epidermal filaments were measured using ImageJ version 1.30 (National Institutes of Health, USA). Setal spatulae were considered to approximate isosceles triangles for the purpose of determining their area. For each measurement taken, averages and standard deviations for each subdigital scale across the sample of six individuals were calculated.

Examination of the distal ends of the filament arrays was carried out by rotating the SEM stubs to bring them into orthogonal orientation with the electron beam. Tips in a 4 μm^2^ rectangle in two to six locations on an individual image were counted and averaged to yield regional estimates of tip density. For one specimen (specimen 6, Table 1), a thorough survey of all pertinent scales of digit IV of the manus and pes was undertaken in this orientation, enabling scale area, filament density, filament tip density, and filament tip area to be correlated. These data were used to compare the proportional contribution of each scale to the overall (seta‐based) adhesive potential of the entire digit.

Estimates of the number of tips per filament could not be determined using ESEM imaging of the longitudinally sectioned digits in either sagittal or ventral (orthogonal) views because the assignment of tips to the crowns of individual filaments could not be reliably accomplished. For these estimates, and for estimates of the spacing of ranks and rows of filaments on each scale, histological procedures (see below) were employed and integrated with information gleaned from SEM observations.

#### 2.3.2 Histology

Manual and pedal digits were removed from an additional specimen of the initial sample of 25 formalin fixed, ethanol‐preserved Tokay geckos (*Gekko gecko*), re‐hydrated through a graded series of ethanol (50% for 2 h, 30% for 3 h) followed by immersion in distilled water for several hours. Decalcification was achieved by immersion in Cal‐Ex (Fisher Scientific) for approximately 48 h, followed by dehydration through a graded ethanol series (30%, 50%, 70%, 95%, and absolute ethanol), and clearing in Safeclear (Fisher Scientific) for 3.5 h. Infiltration in a vacuum oven at 60°C with Paraplast Plus (Kendall) in three consecutive 1‐h baths followed, prior to embedding in Paraplast Plus.

Blocks were frontally sectioned at 10 μm on a rotary microtome; the sections floated onto warmed, albumin‐coated slides and positioned manually, dried on a slide warmer for 2 days, stained with hematoxylin and eosin (Humason, 1979), mounted in DPX (BDH Chemicals) and examined using a Nikon 550S microscope. Measurements of the epidermal filaments were taken for scales near the distalmost and proximalmost extremities of each of the OB topographical regions (two or three regions, depending upon the digit in question) (Figure 2), thereby automatically encompassing both TB regions (Figure 2). Stalk diameter and the basal area of stalk groupings were measured from digital images using ImageJ version 1.30 (National Institutes of Health, USA). The distance between individual filament tetrads (or their equivalent when the pattern was less regular) within rows and between tetrads in ranks (Figure 5) was measured, along with the mediolateral and proximodistal dimensions of the tetrad clusters (Figure 5). Counting the number of stalks in a 1000 μm^2^ area enabled estimation of stalk density per unit area. The number of tips per individual filament could not be accurately counted in the SEM images (see above), so the estimated number of tips per seta was obtained by permutating the results of tip density estimates and stalk density estimates.

**FIGURE 5:**
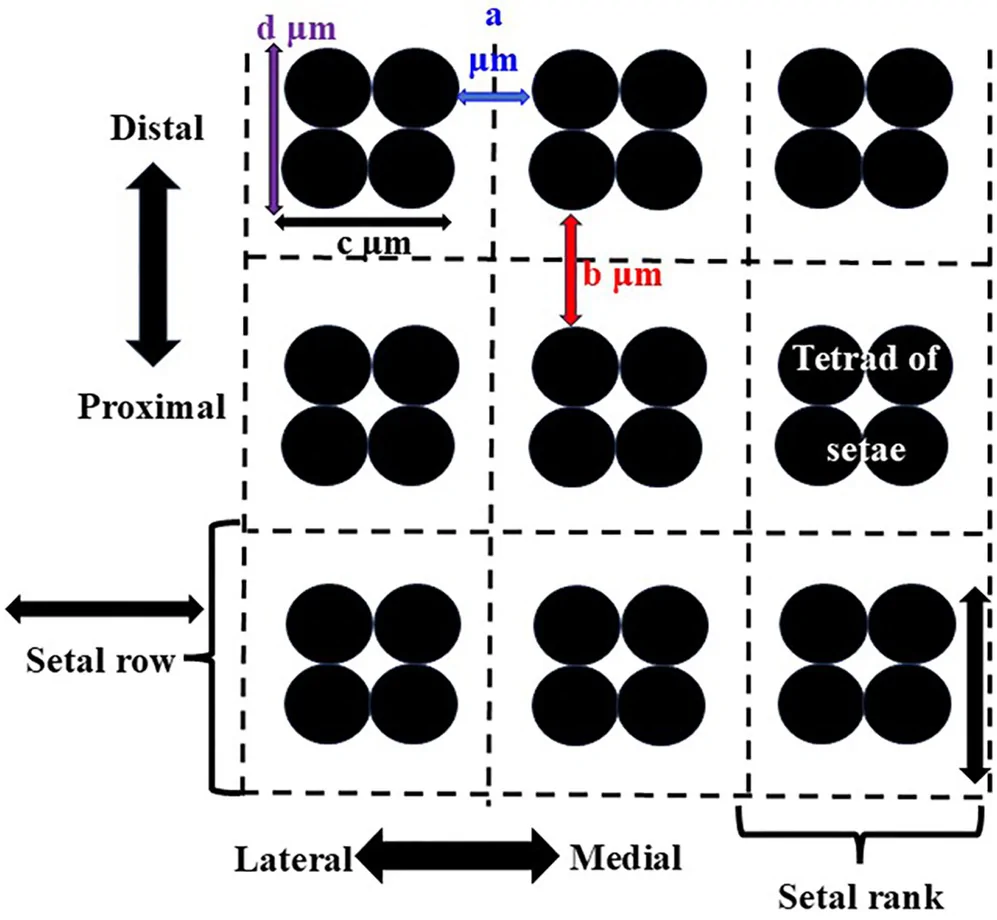
Method employed for assessing filament stalk dimensions and density of *Gekko gecko* using frontal histological sections of filament fields. The example here depicts setae clustered into tetrads, but other filament aggregations also occur (see text for details). Filaments are arranged in mediolateral rows and proximodistal ranks. Measurement was made, in μm, of the distance between filament groupings (here tetrads) in adjacent ranks (measurement *a*) and adjacent rows (measurement *b*); mediolateral width of filament groups (measurement *c*) at their base; proximodistal length of filament groups at their base (measurement *d*). Measurement *c* × measurement *d* yields the basal area of stalk clusters in μm^2^. Measurements (*a* + *c*) × (*b* + *d*) yield the basal area of the stalk domains in μm^2^. These data were used to calculate stalk density per mm^2^ and to assess the approximate number of tips per filament (see text for details).

## 3 RESULTS

### 3.1 Digit gross anatomy

#### 3.1.1 Scale dimensions

Although the number of OB regions differs between digits (Figure 2), scale counts for the distal region are similar for the same digit among all animals studied (Table 2). Greater differences occur between the proximal regions of digits that have an intermediate region and those that do not (Table 2). Digits with only a proximal and a distal region have more scales associated with the proximal region than those having an intermediate region. Occasionally, digits bearing an intermediate region exhibit no enlarged proximal subdigital scales (Table 2).

**TABLE 2:** Scale count ranges and average total areas by osteologically based (OB) digital region (proximal, intermediate, and distal) for the left and right manus and pes of *Gekko gecko* for all individuals examined (*n* = 6).

	Proximal region scale count range	Average proximal region area L/R mm^2^	Intermediate region scale count range	Average intermediate region area L/R mm^2^	Distal region scale count range	Average distal region area L/R mm^2^
Manus						
Digit I	5–7	6.47/7.25	N/A	N/A	9–11	10.09/9.02
Digit II	5–8	9.14/8.79	N/A	N/A	9–10	10.37/10.47
Digit III	2–5	3.19/4.04	5–6	10.02/9.74	8–10	9.73/9.97
Digit IV	0–4	1.33/1.71	7–9	13.01/13.08	9–10	9.67/9.95
Digit V	3–6	9.06/9.72	N/A	N/A	9–10	11.25/11.52
Pes						
Digit I	4–8	8.00/8.57	N/A	N/A	10–11	13.50/12.66
Digit II	4–8	10.66/11.05	N/A	N/A	9–11	14.09/13.29
Digit III	2–6	3.30/3.85	6–7	13.06/12.67	9–10	13.48/13.43
Digit IV	0–4	2.53/1.87	8–11	17.93/17.49	9–11	12.68/12.31
Digit V	0–3	4.04/3.79	5–7	13.32/14.48	9–11	12.40/12.64

*Note*: Scale count numbers per individual are sometimes asymmetrical for the manus and/or pes. N/A indicates not applicable because of the absence of an intermediate region in those digits.

Subdivision of digits into a TB LDT region distally and a basal region proximally (Figure 2) reveals similar scale counts for all six individuals for the basal and LDT regions of each digit (Table 3). Scale counts within the LDT region are similar among the digits within the same autopodium (manus and pes) but are higher for the pedal digits (Table 3).

**TABLE 3:** Scale count ranges and average total areas by tendon‐based (TB) digital region (basal and LDT) for the left and right manus and pes of *Gekko gecko* for all individuals examined (*n* = 6).

	Basal region scale count range	Average basal region area L/R mm^2^	LDT region scale count range	Average LDT region area L/R mm^2^
Manus				
Digit I	2–4	2.31/3.03	12–14	14.24/13.24
Digit II	2–5	4.08/3.81	12–13	15.43/15.44
Digit III	3–6	3.19/4.04	13–15	19.75/19.70
Digit IV	3–6	4.91/5.93	13–15	18.65/17.95
Digit V	2–5	4.96/4.40	12–13	16.49/17.00
Pes				
Digit I	1–5	2.81/3.21	13–14	18.69/18.70
Digit II	1–5	4.82/5.01	12–14	19.93/20.21
Digit III	2–6	3.30/4.02	16–17	26.54/27.15
Digit IV	3–7	7.24/6.95	14–17	24.64/25.23
Digit V	0–4	2.90/2.44	15–18	25.72/27.18

*Note*: Scale count numbers per individual are sometimes asymmetrical for the manus and/or pes.

Scale length, width, and area exhibited similar patterns and measurements across all digits among both sexes and the size range of geckos examined. Measurements are, therefore, presented as averages for all geckos (Figures 6, 7, 8).

**FIGURE 6:**
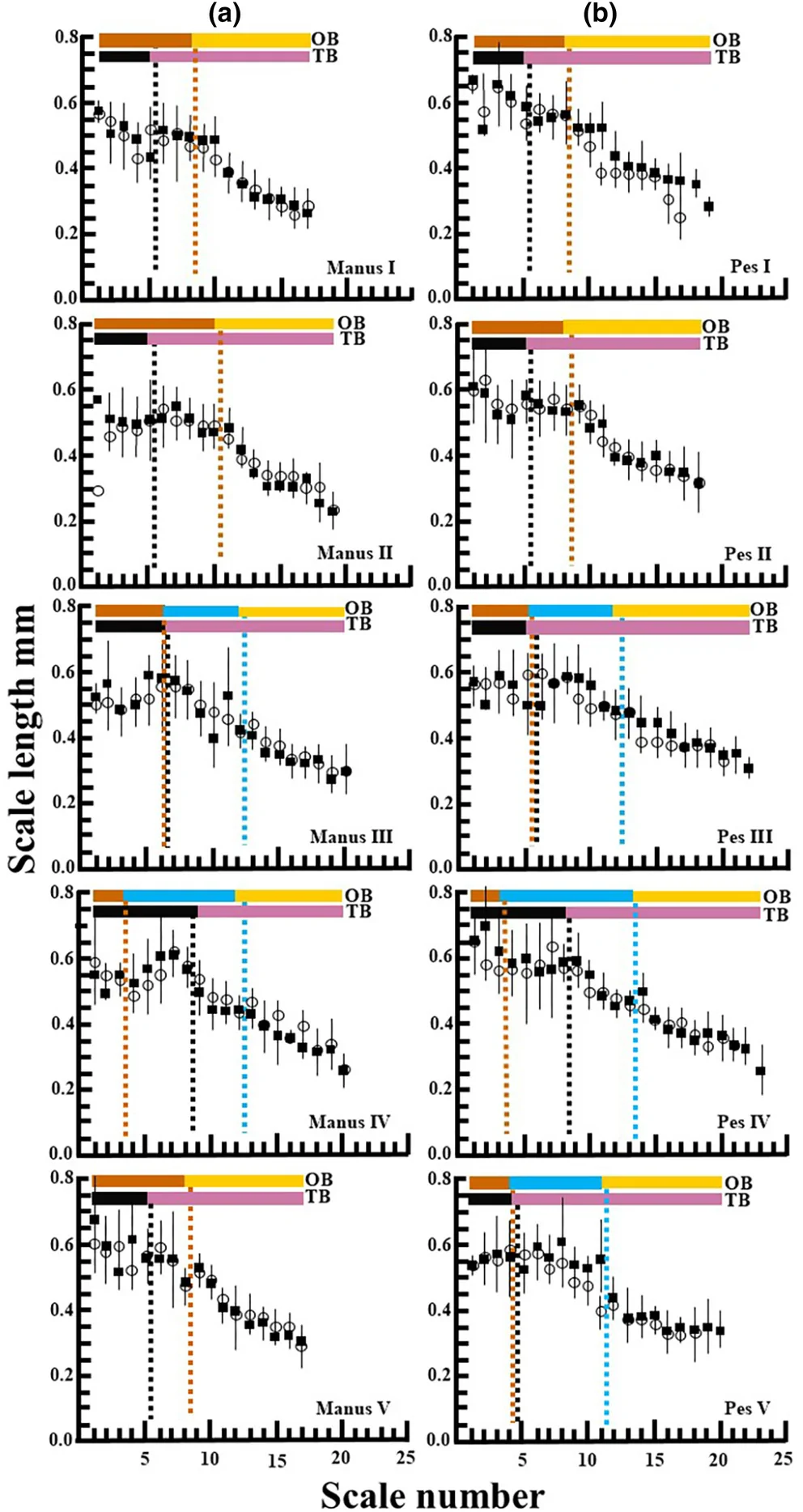
Plot of average subdigital scale lengths for *Gekko gecko* (*n* = 6 except for manus digit V where *n* = 5) along the median transect line (Figures 1b and 4a) for all digits of the manus (column a) and pes (column b). Subdigital scales are numbered in reverse order from proximal to distal (see Figure 3a for a depiction of this convention), The number of included scales varies from digit to digit (see Figure 1b) between and within individuals. Open circles represent right digits, filled squares represent left digits. Error bars indicate standard deviation (these mostly overlap for the left and right measurements). Dotted vertical brown lines represent the interscale boundary between the osteologically based (OB) proximal (brown bar) and either distal (yellow bar) or intermediate (blue bar) zones, and dotted blue lines the interscale boundary between the OB intermediate and distal zones (depending upon digit—see text for explanation). Dotted vertical black lines represent the interscale boundaries between the tendon‐based (TB) basal (black bar) and LDT (lateral digital tendon—lilac bar) zones (equally applicable to all digits—see text for explanation).

Scale lengths along the median transect (Figure 1b) decrease modestly from proximal to distal, then increase slightly before declining more sharply distally (Figure 6). The greatest degree of variation in length occurs among the more proximally situated scales (Figure 6) because alignment of the series of subdigital scales employing the distalmost scale (see above for explanation) renders the more proximal regions less equivalent between individuals. This is reflective of variation in the total number of subdigital scales between both digits and individuals (Figure 6; Tables 2 and 3).

Examination of trends within the OB regions reveals that the longest scales occur in the intermediate region, if present, or, when absent, in the proximal region (Figure 6). Scales of the distal region decrease in length from proximal to distal (Figure 6).

Trends for the TB regions show that the scales exhibit a more uniform pattern of decline in length proximodistally than is evident for the OB regionalization (Figure 6). The TB basal region exhibits a more variable pattern, especially for those digits that possess three OB regions (Figures 2 and 6).

The width of subdigital scales increases from the base of the digit to its midpoint and then decreases again further distally (Figures 1 and 7). For digits that lack an OB intermediate region (Figure 2), the width of scales in the proximal region increases from proximal to distal (Figure 7). For such digits, the boundary between the proximal and distal regions occurs where the scales are the widest (Figure 7). For digits where an intermediate region is present, the widest scales occur there (Figure 7).

**FIGURE 7:**
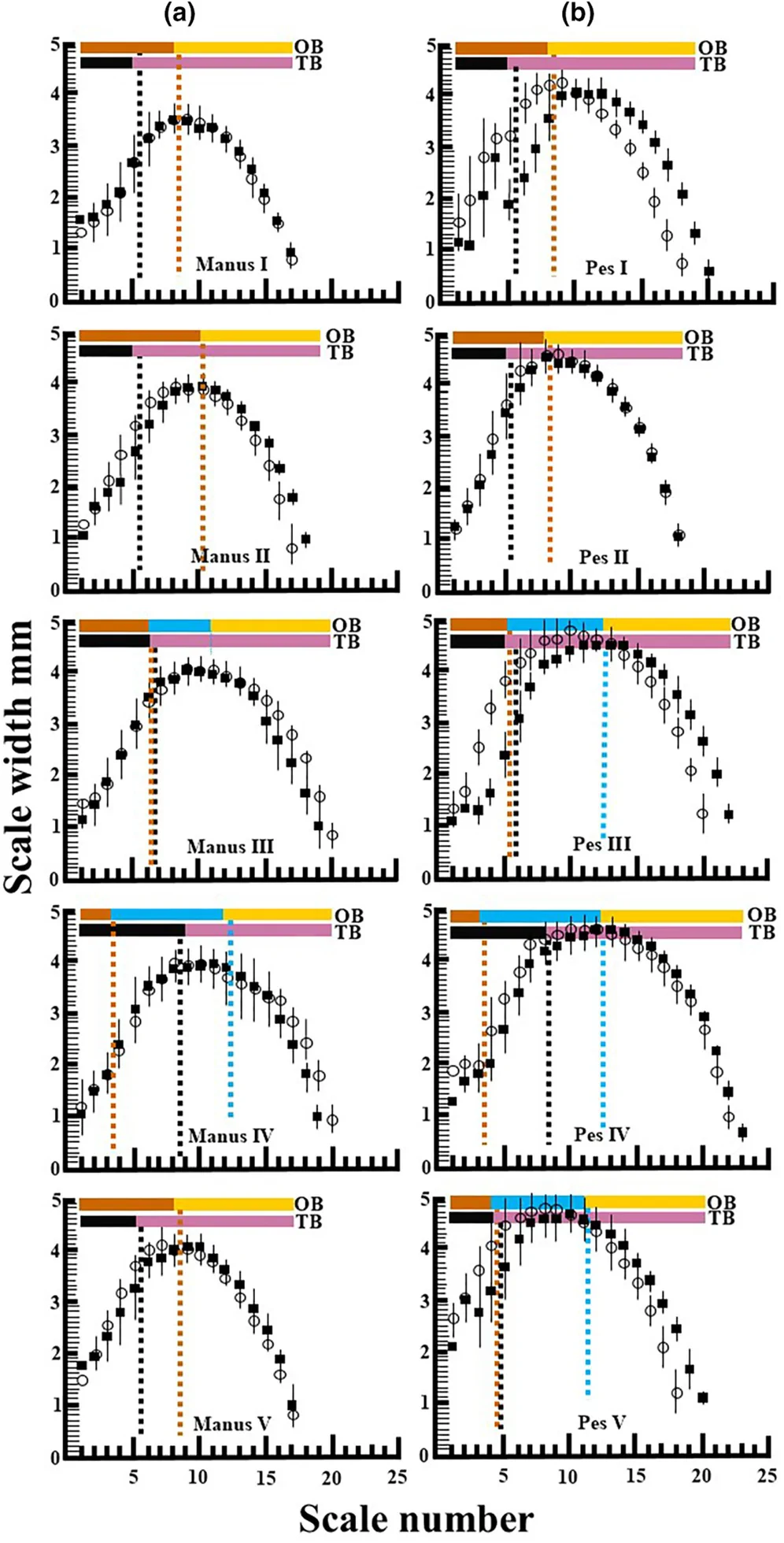
Plot of average subdigital scale widths for *Gekko gecko* (*n* = 6 except for manus digit V where *n* = 5) along the median transect line (Figures 1b and 4a) for all digits of the manus (column a) and pes (column b). Conventions for scale numbering, left and right digits, error bars and depiction of boundaries between digital zones are identical to those described for Figure 6.

When considering the two TB regions (Figure 2), the scales of the LDT region continue to increase in width beyond the greatest width of the basal scales, reaching a peak at the midpoint of the length of the digit (Figure 7). Beyond this point, scale width declines steadily toward the distal extremity (Figure 7).

The area of individual subdigital scales is greatest for scales within the OB proximal region (Figure 8) for digits in which an intermediate region is absent (Figure 2), and for the intermediate region when it is present (Figure 8). The distalmost scales have areas smaller than those of the proximalmost ones (Figure 8). Scale counts within the OB basal region tend to be more variable among digits, and thus the overall area of the basal region is also variable among digits (Table 2).

**FIGURE 8:**
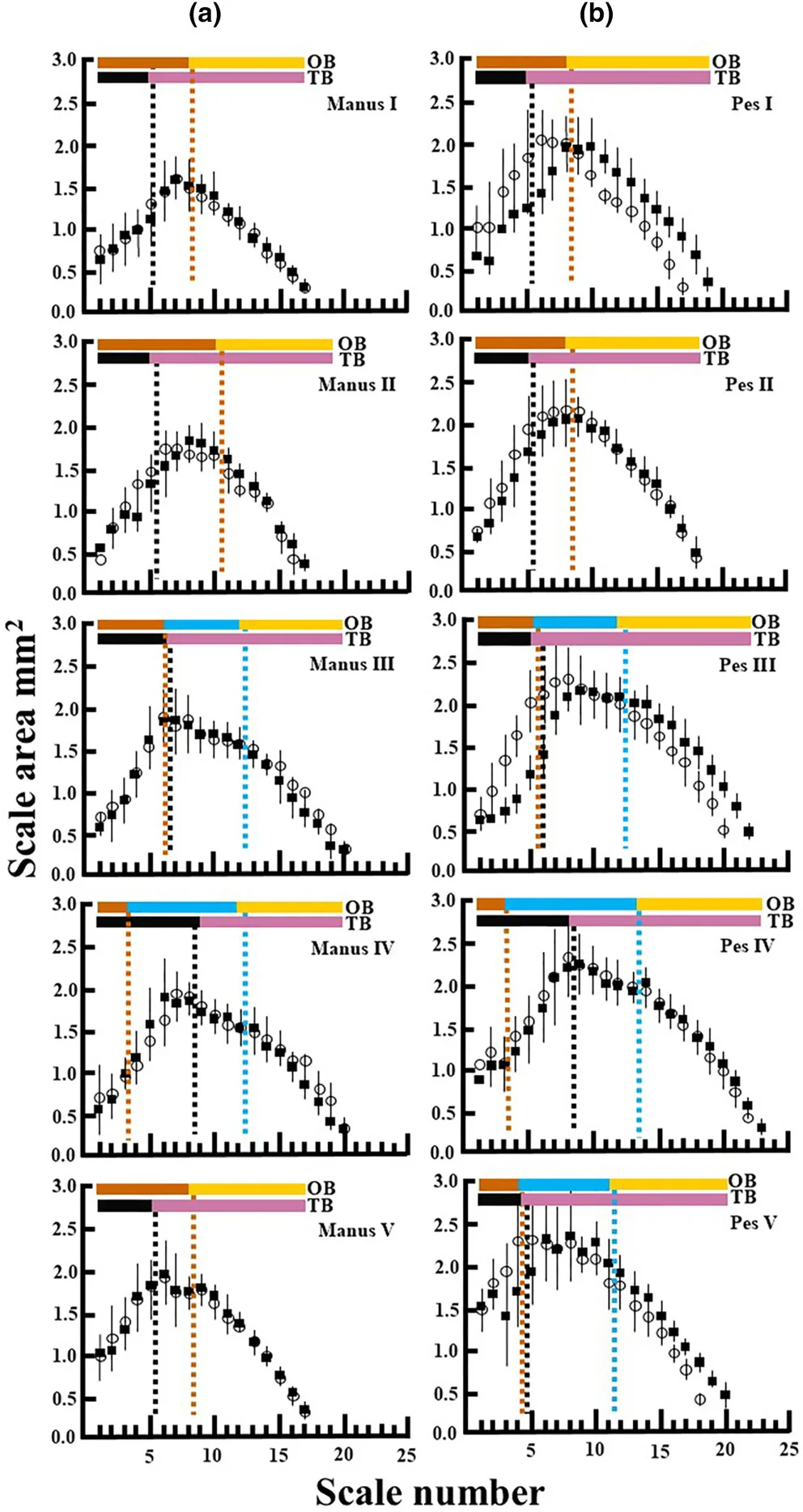
Plot of average subdigital scale areas for *Gekko gecko* (*n* = 6 except for manus digit V where *n* = 5) along the median transect line (Figures 1b and 4a) for all digits of the manus (column a) and pes (column b). Conventions for scale numbering, left and right digits, error bars, and depiction of boundaries between digital zones are identical to those described for Figure 6.

Except for manual and pedal digit IV, where the distalmost scales of the basal region have the greatest area, the TB LDT region (Figure 2) incorporates the scales with the greatest area (Figure 8). Digits with higher scale counts, such as pedal digits III, IV, and V, tend to have a larger overall TB LDT area (Table 3).

Total areas for the OB proximal, intermediate, and distal regions, and the TB basal and LDT regions are consistently greater for the pes versus their structural equivalents of the manus (Tables 2 and 3).

Peterson and Williams (1981) and Bauer and Good (1986) proposed methods for determining which subdigital scales define the proximal boundary of the toe pad when attempting to calculate its area (Figure 1b; Table 4). Both approaches incorporate more scales in the nominal toe pads than those subdividing the digits on osteological or tendinous grounds (see above) (Figures 1b and 2), either including all subdigital scales with a width‐to‐length ratio greater than one (Peterson & Williams, 1981) or a width greater than two and a half times the average palmar scale width (Bauer & Good, 1986). Both approaches employ scale dimensions alone to circumscribe the toe pads. Application of these methods to our sample of Tokay geckos revealed similar results. For all but 18 of the 120 digits inspected (Table 5), application of the Peterson and Williams (1981) criterion led to the inclusion of an extra scale (two for one digit) compared with those included when applying the Bauer and Good (1986) criterion (Figure 1b). In instances where an extra scale was identified, differences between the total measured subdigital areas were minute (Table 5). Application of both methods occasionally incorporated scales underlying the distal extremity of the respective metapodial (Figures 1b and 2).

**TABLE 4:** The average width of scales carried on the palmar and plantar surfaces of all six individuals of *Gekko gecko* examined.

Identification number	Average palmar (manus)/plantar (pes) scale width (mm)	2.5× the average width of the palmar (manus)/plantar (pes) scales (mm)
02	0.38 palmar	0.95 palmar
	0.45 plantar	1.13 plantar
03	0.42 palmar	1.04 palmar
	0.49 plantar	1.24 plantar
06	0.42 palmar	1.04 palmar
	0.55 plantar	1.38 plantar
07	0.38 palmar	0.96 palmar
	0.46 plantar	1.15 plantar
11	0.36 palmar	0.89 palmar
	0.44 plantar	1.09 plantar
16	0.40 palmar	1.00 palmar
	0.47 plantar	1.17 plantar

*Note*: Bauer and Good (1986) included all scales >2.5 times the width of average palmar/plantar scales in the subdigital pad.

**TABLE 5:** Comparisons between scale counts and total pad area between the two available methods for identifying proximally located scales for inclusion into the confines of the toe pad.

Identification number	Limb	Digit	Peterson and Williams (1981)		Bauer and Good (1986)	
			Total scale count	Total pad area (mm^2^)	Total scale count	Total pad area (mm^2^)
02	L manus	III	18	18.42	17	18.08
02	L pes	III	20	27.62	19	27.06
02	R manus	IV	20	23.67	19	23.16
02	R pes	I	16	18.79	15	18.38
03	L manus	II	17	21.21	16	20.71
03	L pes	I	19	24.35	17	23.11
03	R manus	IV	20	24.32	19	23.92
06	R pes	III	18	30.77	17	30.21
07	L pes	III	20	34.32	19	33.59
11	L pes	III	22	28.49	21	27.88
11	R pes	III	20	30.23	19	29.81
16	L manus	I	16	17.76	15	17.31
16	L manus	III	19	24.33	18	23.82
16	L manus	IV	17	23.58	16	22.53
16	R manus	II	16	20.26	15	19.84
16	R manus	IV	19	22.74	18	22.26
16	R pes	I	16	20.92	15	20.29
16	R pes	IV	20	28.99	19	28.32

*Note*: Comparisons are only reported for instances in which differences in the number of scales identified by both methods are evident.

#### 3.1.2 Toe pad dimensions

##### The osteologically based (OB) approach

For manual digits, the summed area of all scales of the OB intermediate region (Figure 2) differs significantly from those comprising the proximal region (*α* < 0.03), with the summed intermediate area always being larger (Figure 9a; Table 2). The total summed areas of the scales of the intermediate region of digits III and IV of the manus are significantly different from each other (*α* < 0.03), with digit IV having a greater area (Figure 9a; Table 2). The summed scale areas of the distal and intermediate regions of manual digit IV are also significantly different from each other (*α* < 0.03), with that of the intermediate area being the larger (Figure 9a; Table 2).

**FIGURE 9:**
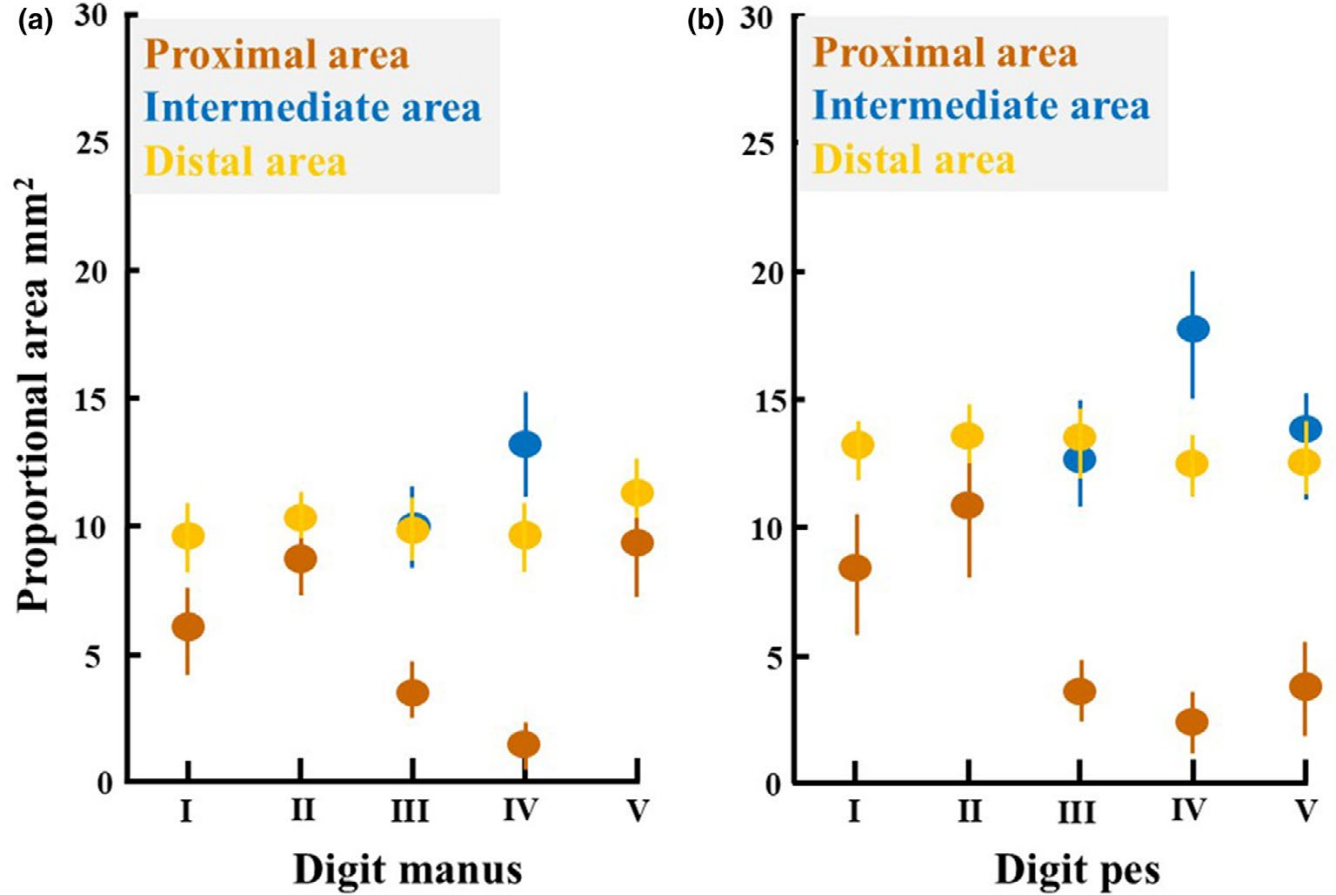
Plot of average total areas for each digit (I–V) for the digit zones of the osteologically based (OB) subdigital subdivisions of *Gekko gecko*. (a) manus, (b) pes (*n* = 6). The proximal, intermediate, and distal digit zones are represented by brown, blue, and yellow, respectively. The intermediate zone is not encountered in all digits (see text for details). Error bars indicate standard deviation.

For the OB pedal digital regions, similar results were obtained. The summed areas of the scales of proximal and intermediate regions of the pedal digits are significantly different (*α* < 0.03), with the intermediate area always being larger (Figure 9b; Table 2). Comparisons between summed intermediate region areas of pedal digits III, IV, and V revealed no significant differences between digits III and V, but digit IV differs significantly from both (*α* = 0.0013 and 0.0015, respectively) in having greater area (Figure 9b; Table 2). The summed scale area of the intermediate region of pedal digit IV is always larger and significantly different from that of the distal region, whereas for digits III and V there are no differences between these two regions (Figure 9b; Table 2).

Comparisons between serially homologous digits of the manus and pes reveal significant differences in summed scale area of the OB intermediate regions (*α* < 0.03), those of the pedal digits always being greater (Figure 9; Table 2).

The contribution of the OB intermediate region to total subdigital area (manus plus pes) reveals very similar proportions among animals studied (Table 6). Although this region is lacking in some digits (Figures 2 and 9), scales within the region contribute between 26.87% and 28.95% of the total subdigital area, representing a greater contribution than the proximal region (Table 6). The intermediate region contributes more to total hindlimb proportional subdigital area (between 30.46% and 33.74%) than forelimb area (between 21.00% and 23.94%) (Table 6), this being associated with the lack of an intermediate region in manual digit V (Figures 2 and 9).

**TABLE 6:** Contribution of the osteologically based (OB) digital regions (proximal, intermediate, and distal) of the combined left and right manus and pes of *Gekko gecko* to the total toe pad areas for all individuals examined (*n* = 6).

Identification number	Total forelimb/ hindlimb toepad area (mm^2^)
02	170.36/249.14
03	214.50/279.84
06	206.14/273.92
07	241.86/331.81
11	188.57/256.17
17	211.02/269.32

Identification number	Proximal region forelimb/hindlimb toepad area (mm^2^)	% contribution of proximal region forelimb/hindlimb to toepad area
02	/43.48	/17.45
03	67.11/61.91	31.29/22.12
06	53.60/43.74	26.00/15.97
07	78.80/77.14	32.58/23.25
11	48.56/50.50	25.75/19.71
17	62.00/50.54	29.38/18.77

Identification number	Intermediate region forelimb/hindlimb toepad area (mm^2^)	% contribution of intermediate region forelimb/hindlimb to toepad area
02	/78.59	/30.46
03	45.05/93.77	21.00/33.51
06	46.42/92.43	22.52/33.74
07	57.90/108.19	23.94/32.61
11	40.39/79.12	21.42/30.89
17	47.41/87.85	22.47/32.62

Identification number	Distal region forelimb/hindlimb toepad area (mm^2^)	% contribution of distal region forelimb/hindlimb to toepad area
02	/129.78	/52.09
03	102.33/124.16	47.71/44.37
06	106.12/137.76	51.48/50.29
07	105.17/146.47	43.48/44.14
11	99.62/132.62	52.83/51.77
17	101.62/130.92	48.16/48.61

*Note*: Digit I of the left manus of specimen 02 lacks area data because of damage.

Summed areas of the scales that compose the OB proximal region vary between digits lacking and possessing an intermediate region (Figure 2). Digits lacking an intermediate region (Figure 2) have greater proximal region summed scale areas (Figure 9; Table 2). The distalmost of the expanded scales within the proximal region of digits lacking an intermediate region exhibits characteristics similar to those of the intermediate region of other digits.

Comparisons of OB summed proximal scale subdigital area reveal significant differences between most digits of the manus and pes, which indicate high variability of the area of this region (Figures 8 and 9; Table 2). Exceptions to this are for manual digits II and V, and pedal digits III and V (Figure 9). In both cases, these digits have the same number of topographical regions (Figures 2 and 9), two for manual digits II and V, and three for pedal digits III and V, and the same number of phalanges (Figure 2).

The OB distal (Figure 2) region contributes a major proportion of the total subdigital area measured (Table 6). The contribution of the distal region to the total area recorded for the forelimbs, hindlimbs, and combined subdigital area is strikingly consistent between animals (Table 6). Despite the pedal distal area being larger than the manual distal area (Figure 9), each contributes to the total subdigital area of the forelimbs and hindlimbs in equal proportions, between 43.48% and 52.83% of manual and pedal total subdigital area (Table 6).

Serial homologue comparisons of the OB distal region (Figure 2) reveal significant differences between the total distal area of manual and corresponding pedal digits (Figure 9; Table 6). Digits of the pes possess a greater scale area in the distal region when compared with equivalent digits of the manus (Figure 9; Table 6), possibly because of the differential length between manual and pedal digits. Pedal digits tend to have absolutely longer phalanges (Figure 2).

##### The tendinously based (TB) approach

For manual digits, the TB division into basal and LDT regions (Figure 2) reveals that the summed scale areas of the LDT regions and basal regions of the same digit are significantly different from each other (*α* < 0.008), with the area of the LDT region being markedly larger (Figure 10a; Table 3). Except for digits I and III (*α* = 0.0036), the areas of the LDT regions of all manual digits are not significantly different from each other (Figure 10a; Table 3). Basal region summed area comparisons between manual digits reveal mixed results as some, but not all, digits are significantly different from each other (digit I and all other digits, digits III and IV [*α* < 0.008]). Digit I carries the smallest basal area, followed by digit III, while digit IV has the largest (Figure 10a; Table 2).

**FIGURE 10:**
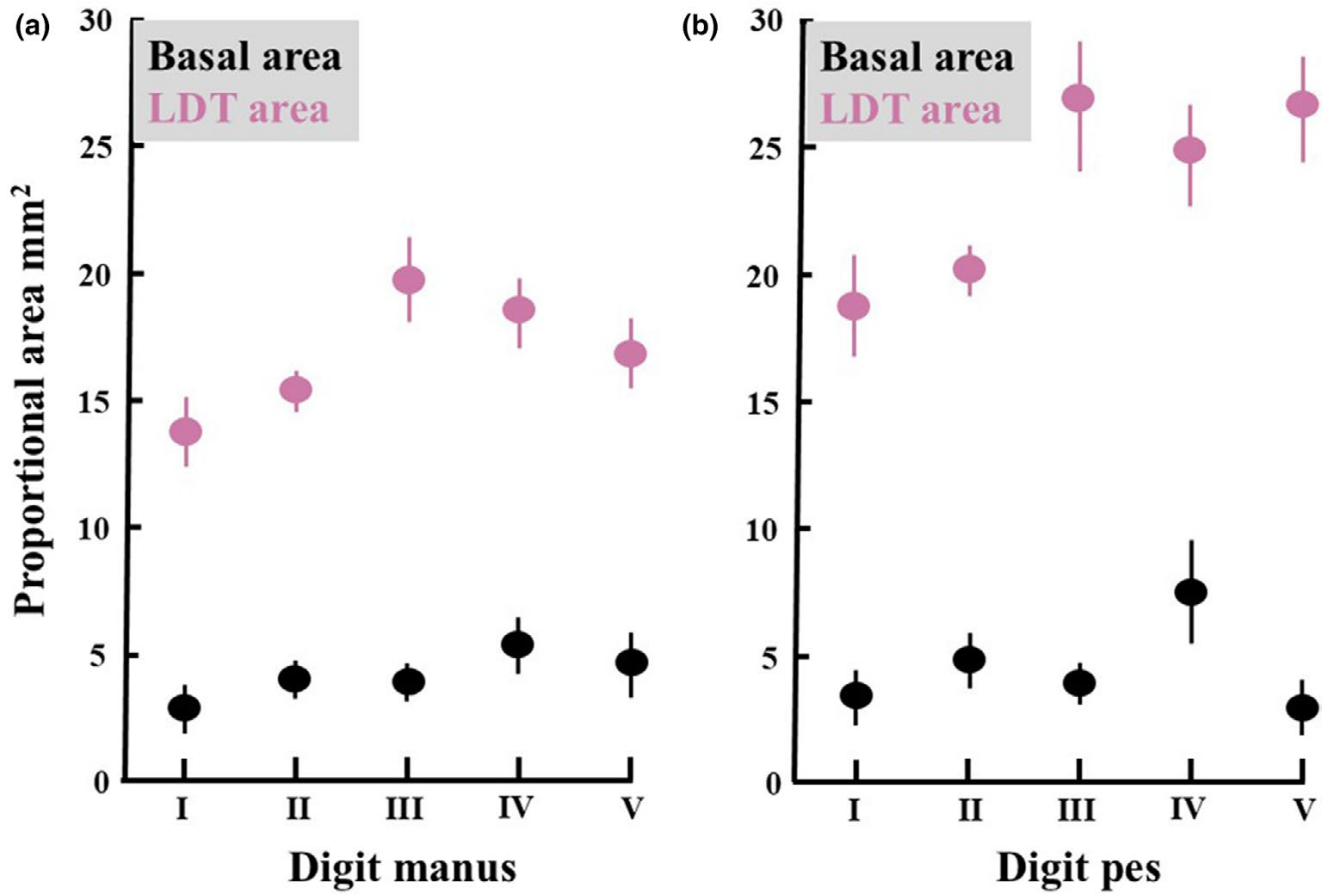
Plot of average total areas for each digit (I–V) for the digit zones of the tendon‐based (TB) subdigital subdivisions of *Gekko gecko*. (a) manus, (b) pes (*n* = 6). The basal and LDT (lateral digital tendon) digit zones are represented by black and lilac, respectively. Error bars indicate standard deviation.

For pedal digits the summed scale areas of the LDT regions and basal regions of the same digit are significantly different (*α* < 0.008), with the area of the LDT region being much larger (Figure 10b; Table 3). Except for digits I and III (*α* = 0.0036) and I and V (*α* < 0.0051), the LDT region is not significantly different between digits (Figure 10b; Table 3). Basal region comparisons between pedal digits reveal variability, with some comparisons being significant and others not. The summed scale area of the basal region of digit I is significantly different from that of digits II and IV (*α* < 0.0008), and the area of the basal region of digit III is significantly different from that of digit IV (*α* < 0.0008). Digits I and V exhibit the smallest basal areas and digits II and IV the largest (Figure 10b; Table 3).

Comparisons of serially homologous digits for the TB subdivisions identified no significant differences between the same region of corresponding digits, except for digit V, where both the LDT and basal regions are significantly different (*α* = 0.0003 and *α* = 0.0006, respectively), with the LDT region being larger and the basal region being smaller for the pes (Figure 10a,b; Table 3). This discrepancy is seemingly related to differences in the phalangeal composition of manual and pedal digit V, with the latter being longer due to the presence of an intermediate phalanx absent from manual digit V (Figure 2). The longer pedal fifth digit thus supports a greater subdigital area. The lack of an OB intermediate region in manual digit V results in a greater proportion of non‐LDT‐associated subdigital scales being incorporated into the TB basal region.

For the TB digital subdivision (Figure 2), the LDT region is the major contributor to total subdigital area of the forelimbs and hindlimbs, ranging between 76.53% and 86.77% (Table 7). The consistency between digits and autopodia of the large percentage contribution of the TB LDT area (Tables 3 and 7) suggests the importance of contact area within this region for the functioning of the adhesive system.

**TABLE 7:** Contribution of the tendon‐based (TB) digital regions (basal and lateral digital tendon [LDT]) of the combined left and right manus and pes of *Gekko gecko* to the total toe pad areas for all individuals examined (*n* = 6).

Identification number	Total forelimb/hindlimb toepad area (mm^2^)	Basal region forelimb/hindlimb toepad area (mm^2^)	% contribution of basal region forelimb/hindlimb to toepad area	LDT region forelimb/hindlimb toepad area (mm^2^)	% contribution of LDT region forelimb/hindlimb to toepad area
02	170.36/249.14	/35.14	/14.10	/214.00	/85.90
03	214.50/279.84	47.51/49.21	22.15/17.59	167.91/230.62	78.28/82.41
06	206.14/273.92	32.28/36.25	15.66/13.23	173.85/237.67	84.34/86.77
07	241.86/331.81	56.77/61.66	23.47/18.58	185.09/270.15	76.53/81.42
11	188.57/256.17	29.36/33.88	15.57/13.23	159.21/222.29	84.43/86.77
17	211.02/269.32	44.25/40.11	20.97/14.89	166.77/229.24	79.03/85.11

*Note*: Digit I of the left manus of specimen 02 lacks area data because of damage.

### 3.2 Digit microscopic anatomy

#### 3.2.1 Scanning electron microscopy

For every digit examined for all geckos, measurements of the filaments carried on the subdigital scales revealed similar patterns and trends. Graphical representations (Figures 11, 12, 13) are, therefore, provided only for digit IV (manus and pes), the longest digit on each autopodium. Average length of the filaments along the median transect increases from proximal to distal (Figure 11a,b). Average basal diameter of the filaments follows a similar trend along this transect, with those of the distal scales having the widest base (Figure 11c,d). The average tip diameter of the filaments also increases from proximal to distal along the digit (Figure 12a,b), and the tips change in form from hooks to spatulae along this trajectory (Figure 12a,b).

**FIGURE 11:**
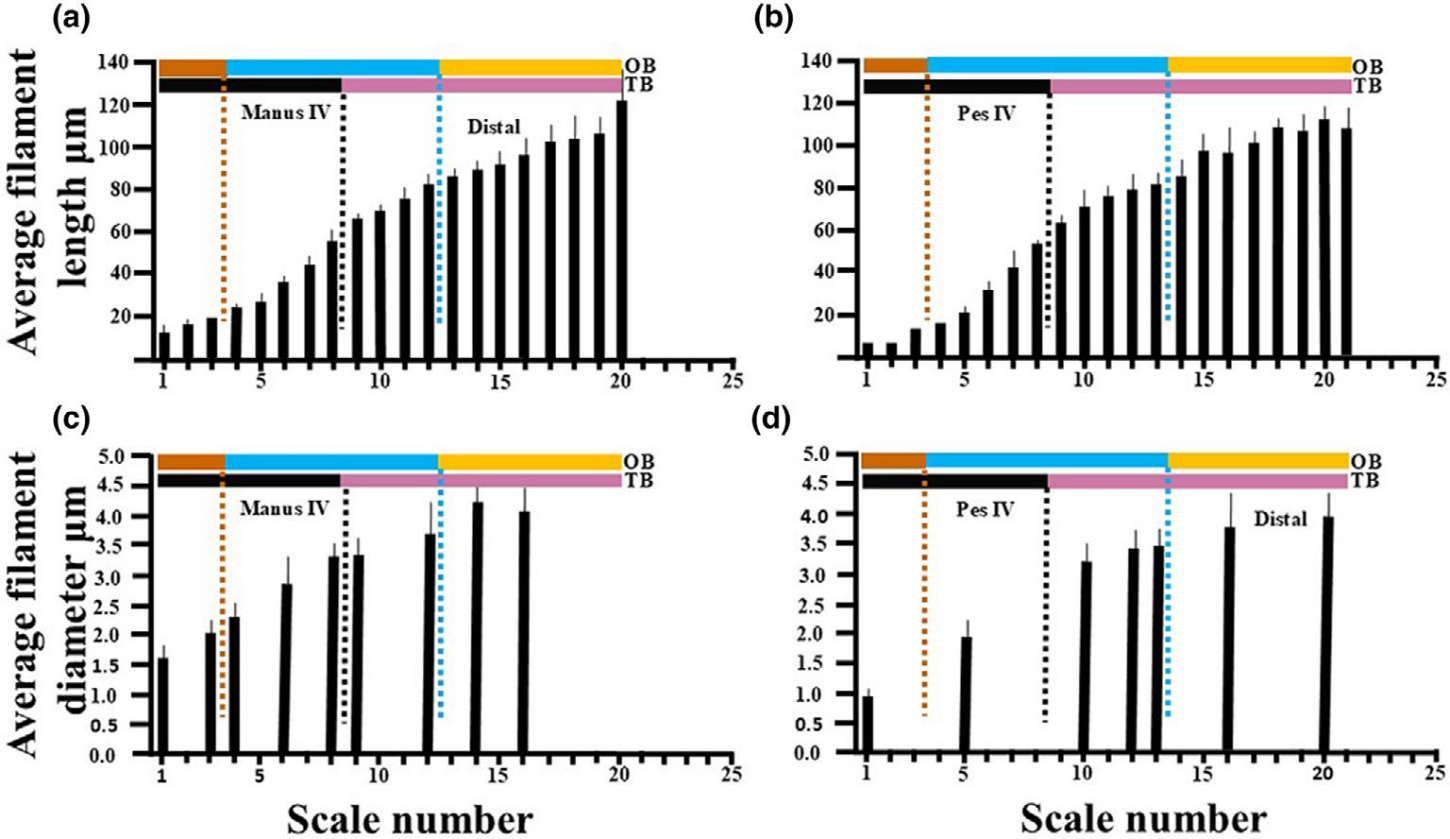
(a, b) Plots of average filament lengths carried on the subdigital scales of left and right digit IV manus (a) and pes (b) of *Gekko gecko* (*n* = 6 except for manus digit V where *n* = 5) along the median transect line (Figures 1b and 4a). (c, d) Plots of average stalk diameters for selected subdigital scales of left and right digit IV manus (c) and pes (d) of *Gekko gecko* (*n* = 4) along the median transect line. Subdigital scales are numbered in reverse order from proximal to distal (see Figure 3a for a depiction of this convention; the actual number of scales varies between individuals). Error bars indicate standard deviation. Colored bars at the top of each panel indicate the extent of the osteologically based (OB) proximal (brown), intermediate (blue), and distal (yellow) zones and the tendon‐based (TB) basal (black) and LDT (lateral digital tendon—lilac) zones. Dotted vertical brown lines represent the interscale boundary between the OB proximal and either distal or intermediate zones, and dotted blue lines the interscale boundary between the OB intermediate and distal zones (depending upon digit—see text for explanation). Dotted vertical black lines represent the interscale boundaries between the TB basal and LDT zones (equally applicable to all digits—see text for explanation).

**FIGURE 12:**
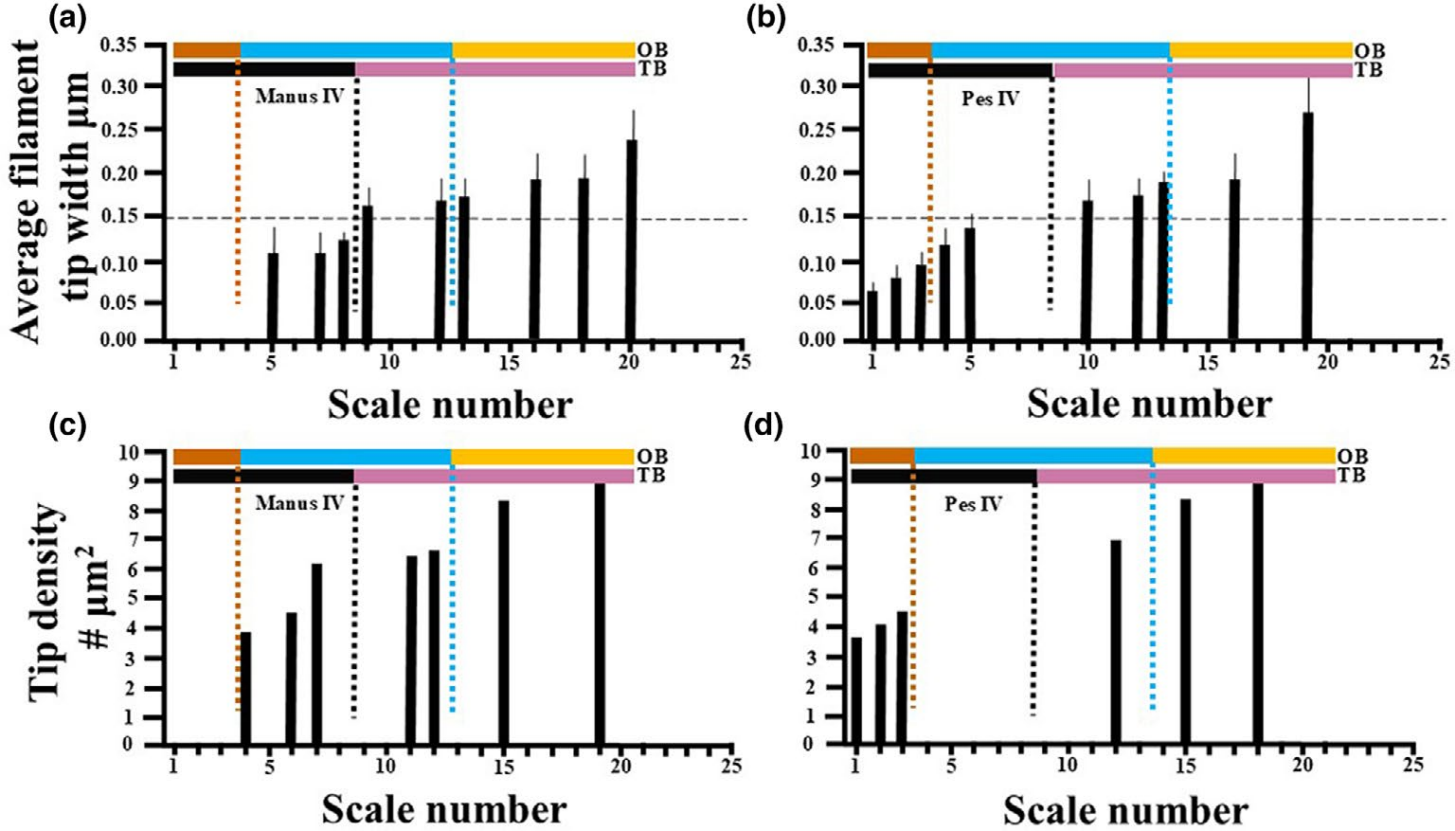
(a) and (b) Plots of average filament tip width carried on selected subdigital scales of left and right digit IV manus (a) and pes (b) of *Gekko gecko* (*n* = 1) along the median transect line (Figures 1b and 4a). The dashed horizontal line on panels a and b indicates a tip diameter of 0.15 μm, below which filaments no longer carry spatulate tips. (c) and (d) Plots of average filament tip density for selected subdigital scales of left and right digit IV manus (c) and pes (d) of *Gekko gecko* (*n* = 1) along the median transect line. Conventions for scale numbering, error bars and depiction of boundaries between digital zones are identical to those described for Figure 11.

Tip densities are highest for the most distal scale and decrease steadily from distal to proximal along the length of the digit (Figure 12c,d; Tables 8 and 9), suggestive that the number of branches per individual filament increases along the length of the digit.

**TABLE 8:** Data for manual digit IV of *Gekko gecko* specimen 6.

Scale # (see Figures 3, 4, 6, 7, 8, 11, 12, 13)	Scale area mm^2^	Estimated tips per mm^2^	Average tip width μm	Average tip area mm^2^	Estimated total tip area per scale μm^2^	Estimated total filament stalks per scale	Density of filament stalks per mm^2^	Estimated average number of tips per stalk	OB divisions	TB divisions
20	0.162				42,000	2592	15,992			
19	0.287	9.0 × 10^−6^	0.24	2.87 × 10^−8^	74,000	4592	15,980	563		
18	0.482				104,000	7712	15,964			
17	1.033		0.19		173,000	16,528	15,999			
16	1.185				198,000	18,960	15,945			
15	1.283	8.29 × 10^−6^	0.19	1.81 × 10^−8^	193,000	20,528	16,007	518		
14	1.417				212,000	22,672	16,097			
13	1.551				233,000	24,816	16,006			
12	1.417	6.50 × 10^−6^	0.15	1.47 × 10^−8^	135,000	22,672	15,893	406		
11	1.607	6.40 × 10^−6^	0.14	1.43 × 10^−8^	147,000	25,712	15,992	400		
10	1.592				146,000	25,472	16,047			
9	1.699				155,000	27,184	16,011			
8	1.778	6.21 × 10^−6^	0.10	1.33 × 10^−8^	144,000	42,672	23,981	259		
7	1.900		0.07		196,000					
6	1.941	4.55 × 10^−6^	0.01	7.0 × 10^−9^	54,000	77,640	39,984	114		
5	1.582				44,000					
4	1.084	3.80 × 10^−6^	0.01	5.8 × 10^−9^	24,000	54,200	49,972	76		
3	0.980				22,000					
2										
1										

*Note*: For some scales the number of filament tips per mm^2^, average tip width in μm, average total filament tip area in mm^2^ were calculated, and these data were employed, in combination with histological data pertaining to filament distribution (Figure 14) to estimate the average number of tips per filament stalk. Estimates of total tip area per scale in μm^2^, total number of filament stalks per scale, and density of filament stalks per mm^2^ were assembled from data collected for this specimen as part of the survey of all six specimens examined. The two columns at the right indicate the assignment of each scale to the zones of the digit as subdivided according to the osteologically based (OB) and tendon‐based (TB) criteria: OB yellow, distal; blue, intermediate; brown, proximal; TB lilac, LDT (lateral digital tendon); black, basal.

**TABLE 9:** Data for pedal digit IV of *Gekko gecko* specimen 6.

Scale # (see Figures 3, 4, 6, 7, 8, 11, 12, 13)	Scale area mm^2^	Estimated tips per mm^2^	Average tip width μm	Average tip area mm^2^	Estimated total tip area per scale μm^2^	Estimated total setal stalks per scale	Density of setal stalks per mm^2^	Estimated average number of tips per stalk	OB divisions	TB divisions
20	0.334				100,000	5344	15,999			
19	0.974				291,000	15,584	16,005			
18	1.213	9.0 × 10^−6^	0.26	3.32 × 10^−8^	362,000	19,408	15,992	563		
17	1.333				398,000	21,328	15,996			
16	1.384				414,000	22,144	16,010			
15	1.630	8.29 × 10^−6^	0.21	2.30 × 10^−8^	311,000	26,080	15,987	518		
14	1.701				324,000	27,216	16,003			
13	1.815				346,000	29,040	16,001			
12	1.920	6.90 × 10^−6^	0.19	1.78 × 10^−8^	236,000	30,720	16,005	431		
11	2.063		0.17	1.53 × 10^−8^	218,000	33,008	16,008			
10	2.112				223,000	33,792	15,983			
9	2.222		0.17	1.40 × 10^−8^	215,000	35,552	15,998			
8	2.269				219,000	54,456	24,015			
7	2.454				237,000					
6	2.828				273,000					
5	2.830				273,000					
4	1.936		0.01	9.20 × 10^−9^	123,000					
3	1.446	4.50 × 10^−6^	0.01	6.90 × 10^−9^	45,000	72,300	50,031	130		
2	1.262	4.10 × 10^−6^	0.004	4.30 × 10^−9^	18,000			103		
1	0.856	3.70 × 10^−6^	0.003	3.20 × 10^−9^	10,000			53		

*Note*: All information is the same as that outlined for Table 8.

Filaments carried on the scales of the OB distal and TB LDT regions (Figures 11, 12, 13) have the characteristics of typical setae (Autumn et al., 2000, 2002; Autumn & Peattie, 2002; Garner & Russell, 2021; Hansen & Autumn, 2005; Maderson, 1970; Ruibal & Ernst, 1965). Within the OB distal region (Figure 2), the average length of individual setae ranges from 120 μm distally to 85 μm proximally (Figure 11a,b), and individual stalk diameter averages from 4.2 μm distally to 3.7 μm proximally (Figure 11c,d). Setal tips within the distal region are spatulate, and their width ranges from 0.28 μm distally to 0.17 μm proximally (Figure 12a,b). The setae are disposed in tetrads (Figures 5 and 14b,c), as previously described (Maderson, 1966; Ruibal & Ernst, 1965; Schleich & Kästle, 1986). The number of rows of setae per scale decreases from proximal to distal within this region, ranging from 25 proximally to 8 distally (Figure 13a,b), reflective of the proximal to distal decrease in length of the scales (Figures 1 and 5). Rows (Figure 5) are harder to distinguish in the proximal region of each scale. Comparisons between the same scales of the corresponding digit of the manus and pes (digit IV in this instance) reveal little difference in filament dimensions at equivalent locations (Figures 11, 12, 13; Tables 8 and 9).

**FIGURE 13:**
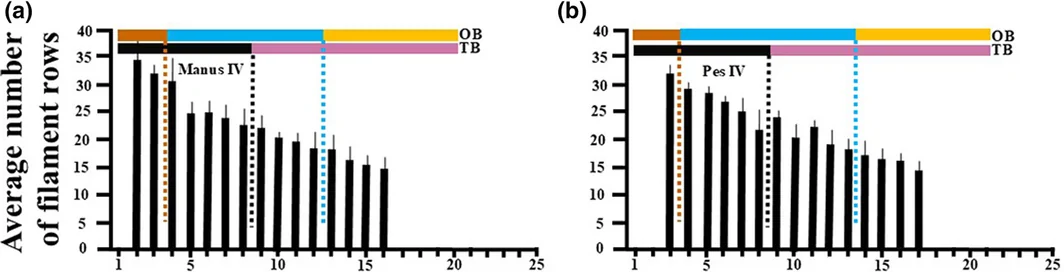
(a) and (b) Plots of average number of filament rows carried on subdigital scales of left and right digit IV manus (a) and pes (b) of *Gekko gecko* (*n* = 6) along the median transect line (Figures 1b and 4a). Data for the distalmost scales and basalmost scales were not obtained. Conventions for scale numbering, error bars, and depiction of boundaries between digital zones are identical to those described for Figure 11.

**FIGURE 14:**
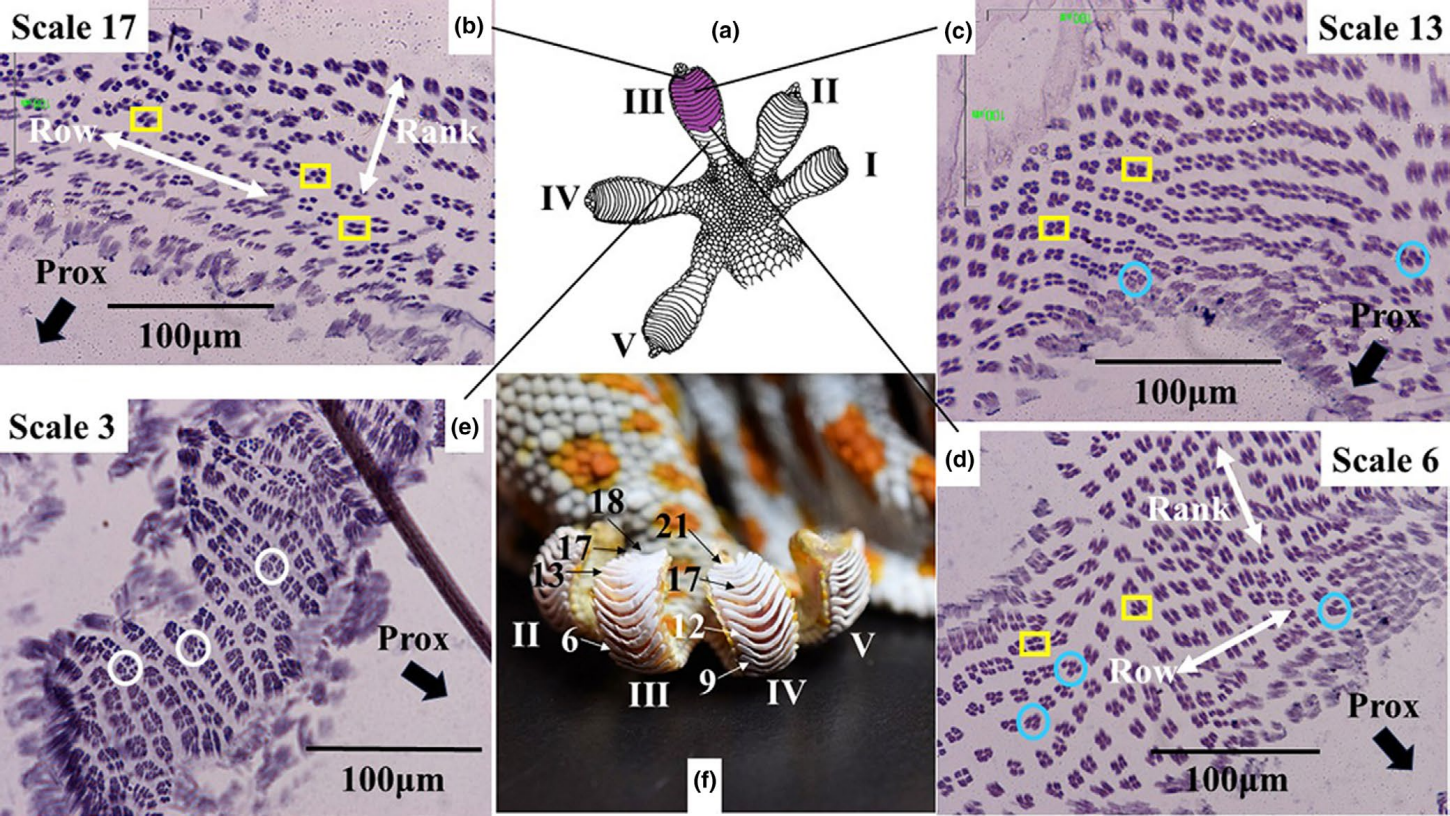
Filament arrangement along the length of pedal digit III of *Gekko gecko* as revealed by frontal sections of the subdigital scales. (a) Ventral view of the pes indicating the location of the filament samples depicted in panels b–e. The subdigital scales with purple fill in panel a collectively comprise the definitive toe pad (consisting of those scales bearing filaments with spatulate tips and being associated with the lateral digital tendons). Measurement of toe pad area that includes expanded scales proximal to these will overestimate toe pad area. For dimensional aspects of the stalk aggregations, see the text and Figure 5. (b) A distal subdigital scale with setal tetrads indicated by yellow boxes. Setal rows and ranks are indicated by double‐headed white arrows. (c) A subdigital scale in the middle region of the definitive toe pad. Setal tetrads are indicated by yellow boxes. Deviations from tetrad associations of stalks and filament bundles are indicated by blue circles. (d) A subdigital scale at the proximal extremity of the toe pad proper. Setal tetrads are indicated by yellow boxes and filament bundles are indicated by blue circles. Ranks and rows of filaments are indicated. (e) A basal subdigital scale with large bundles of filaments indicated by white circles and an absence of tetrads of filaments. For all panels “prox.” indicates the proximal region of the depicted scale. (f) Right manus of *Gekko gecko* with the digits (indicated by Roman numerals) in the fully hyperextended configuration, showing the subdigital scales (scansors) that are raised clear of the substratum. The scales are numbered from distal to proximal using the same convention as in Figure 3. Several enlarged scales (lamellae) lie proximal to the hyperextended portion of the digit and do not participate in the complex biomechanics of the toe pad proper.

There is considerable dimensional variation among the filaments carried on the scales included in the OB intermediate region. The trend in diminution in length of the filaments observed in the OB distal region continues throughout the intermediate region (Figure 11a,b), ranging from an average of 82 μm distally to 18 μm proximally. Basal diameters (Figure 11c,d) range from an average of 3.7 μm distally to 2.3 μm proximally. From distal to proximal in the intermediate region the tips of the filaments trend from spatulate distally to more attenuated and hooked more proximally, ranging in average width from 0.19 to 0.12 μm (Figure 12a,b). The transition from setae to branched prongs is quite abrupt within this region (Figure 12a,b). The average number of rows of filaments carried on the scales of the intermediate region decreases from 31 proximally to 18 distally (Figure 13a,b). On the most proximal scales of the intermediate region, rows are difficult to distinguish, and the filaments are not consistently arranged in tetrads (see below).

The scales of the OB proximal region carry filaments averaging in length from 20 μm distally to 8 μm proximally (Figure 11a,b), with shaft diameters (Figure 11c,d) averaging from 1.9 to 0.78 μm along the same trajectory. The distalmost filaments of this region have an average diameter of 2.0 μm distally and 0.9 μm proximally (Figure 11c,d), and their tips are relatively sparsely branched and attenuated, these filaments being branched prongs. Although such outgrowths may possibly participate in adhesive and/or friction‐enhancing interactions (Khannoon et al., 2014; Spinner et al., 2014), it is unlikely that they can generate sufficient adhesive force on their own to permit whole‐animal adhesion to vertical, low‐friction surfaces (Garner & Russell, 2021; Russell et al., 2015).

For digits lacking an OB intermediate region (digits I, II manus and pes, and manual digit V), filaments of the proximal region display similar characteristics to those of the intermediate and proximal regions described above.

When the digits are divided into the TB LDT and basal regions (Figure 2), the sets of dimensions documented above are subdivided differently. The greatest length of the filaments of the LDT region is, of course, the same as that for the OB distal region (Figure 11a,b). At their shortest, filaments of the LDT region average about 62 μm (Figure 11a,b). The boundary between the LDT and basal region is marked by filament minimum diameters markedly greater than those of the OB proximal region, falling within the range of the OB intermediate region (Figure 11a,b). There is a marked difference in tip width and tip density at the boundary between the TB LDT and basal zones (Figure 12a–d). The LDT region is characterized by all its filaments bearing spatulate tips with a width of 0.15 μm or greater (Figure 12a,b). Proximal to this, tips do not have the spatulate morphology typical of setae (Garner & Russell, 2021) and the filaments carried on the basal region (and the proximal part of the OB intermediate region) (Figure 12a,b) qualify as branched prongs (Garner & Russell, 2021) of varying morphology. Such filaments have not been empirically demonstrated to be involved in adhesive interactions with the substratum, although friction‐enhancing attributes of similarly structured filaments have been demonstrated for chameleons (Khannoon et al., 2014; Spinner et al., 2014).

#### 3.2.2 Histology

Frontal sections through the fields of filaments of digit III of the right manus and pes of *Gekko gecko* reveal that those carried on the scales of the TB LDT region (Figure 2) are arranged in fours (tetrads; Figures 5 and 14b–d), whereas those on the more proximal enlarged subdigital scales (Figure 1b) are arranged in larger groups (Figure 14e). As noted above, there is a continuum of increasing filament stalk diameter along the length of the digit from proximal to distal (Figure 11a,b).

The filaments adorning the scales of the more distal parts of the TB LDT region (Figure 14b), as seen in frontal section, average between 3.4 and 5.0 μm in diameter, similar to the dimensions recorded from SEM images (Figure 11c,d). The area occupied by the base of each tetrad stalk cluster (Figure 5) ranges from 68.4 to 98.6 μm^2^, and averages 79 μm^2^. Each row of tetrads is spaced (Figure 5) an average of 4.88 μm from neighboring rows, and ranks (Figure 5) are spaced on average 3.64 μm from neighboring ranks. There are 4000 tetrads per mm^2^ or 16,000 stalks per mm^2^. Occasionally, stalks are associated in threes or fives, as opposed to fours.

Stalks carried on scales toward the middle of the LDT region (Figure 14c) measure between 1.4 and 3.8 μm in diameter. The area of the base of each tetrad (Figure 5) ranges between 62.7 and 96.7 μm^2^, averaging 80.6 μm^2^. Although the diameter of filaments increases from proximal to distal throughout the TB LDT region, the basal area of the tetrad associations remains consistent. Deviations from tetrad filament associations are more frequent on more proximally located scales (Figures 4e and 14c) in the TB LDT region (those not associated with the penultimate phalanx) than on its more distally located scales.

The proximalmost scale of the TB LDT region (Figure 14d) carries both tetrads and groups of filaments composed of more stalks (up to 6), constituting filament bundles. Rows of tetrads and filament bundles are spaced an average of 5.81 μm from one another, and ranks are separated on average by 4.04 μm from neighboring ranks. There are 4000 filament groupings per mm^2^ on these scales. As a result of the smaller diameter of each individual stalk and the greater number of filaments per group, there are approximately 24,000 filaments per mm^2^ carried on the most basal scale of the TB LDT region (Tables 8 and 9), resulting in a higher filament density than observed on more distally located scales.

Scales of the TB basal region of the digits carry stalks averaging from 0.9 to 1.9 μm in diameter (Figure 11c,d). Among the scales of the basal region, filaments are clustered in larger bundles than observed among the scales of the LDT region (Figure 14e), with the composition of each bundle ranging from 6 to 22 stalks. The distally located scales of the TB basal region carry bundles of 6–15 stalks with a density of approximately 4000 bundles per mm^2^, corresponding to anywhere from 24,000 to 60,000 filaments per mm^2^ (Tables 8 and 9). On more proximally located scales of the TB basal region, each bundle of filaments includes between 10 and 22 stalks, with an average of 16. Bundles are grouped together into more inclusive associations to form clusters of bundles. These clusters have an average spacing of 4.43 μm between rows and 4.75 μm between ranks. There are approximately 3000 clusters per mm^2^.

Collectively, these results indicate that only the scales comprising the TB LDT region have filaments that are consistently amalgamated into tetrads (Figure 14b–d) and terminate in spatulate tips (Figure 12a,b), thereby qualifying as setae (Garner & Russell, 2021). Filaments carried on the scales of the TB basal region (and therefore on the more proximal scales of the OB intermediate region of manual digits III and IV and pedal digits III, IV, and V) are branched prongs, with flattened but non‐spatulate tips (Figure 12a,b), and are aggregated into less regularly arranged bundles and clusters (Figure 14e).

Overall, the data for the subdigital scales reveal that from proximal to distal along the digits the filaments pass through a series of dimensional configurations that grade into the setal form carried by the scales that constitute the definitive toe pad, which is coincident with the TB LDT region. The series begins proximally with short stalks that carry relatively few, narrow tips (branched prongs) (Tables 8 and 9). Proceeding from proximal to distal, with each subsequent scale, the stalks become wider, longer (Figure 11a–d) and terminate in a greater number of tips (Figure 12c,d; Tables 8 and 9). The series also passes, once the toe pad proper is reached, through a transition from narrow to broader spatulate tips (Figure 12a,b; Tables 8 and 9), indicating that the dimensions of the spatulae are associated with the degree of setal branching (Tables 8 and 9), with the tips becoming wider as tip density increases (Figure 12a–d; Tables 8 and 9). “Optimal” spatular width (Geim et al., 2003), a condition difficult to justify in light of the current observations, simply represents the upper end point of an integrated changing pattern of expression of the filaments along the length of the digit.

## 4 DISCUSSION

Two quasi‐quantitative methods for identifying the proximal boundary of toepads have been advanced: incorporation of those subdigital scales that have a width‐to‐length ratio greater than one (Peterson & Williams, 1981), or those that have a width greater than two and a half times that of the average palmar scale (Bauer & Good, 1986) (Figure 2b). Neither of these define the toe pad other than by external scale dimensions. Comparisons of these methods yielded similar results, with differences in estimated toe pad area being minute (Table 5) for the few instances in which any variation in scale inclusion was evident. Application of both methods revealed that expanded scales sometimes extend proximally beyond the first phalanx (Figure 2) to lie ventral to their respective metapodial. Such approaches do not incorporate information about the integumentary microarchitecture present on subdigital scales, which is critical for determining their adhesive capabilities. Furthermore, considering the entire area beneath the digit as constituting the toepad (Beuttell & Losos, 1999), which the methods of both Peterson and Williams (1981) and Bauer and Good (1986) essentially do (Figure 1b), results in an overestimation of toe pad area when the distribution of adhesive setae is considered (Figure 12a,b). All these approaches incorporate scales in the proximal/basal regions of the digits that lack setae. The area of these components (Figures 9 and 10) is highly variable between digits, seemingly at odds with the qualities expected for an adhesive system that is characterized by distributed control (Song et al., 2020).

Anatomically based criteria for determining the extent of the toe pads fare better but differ in their effectiveness. The area of the OB distal region advocated by Rothier et al. (2022) (Figure 2) accounts for a considerable proportion of the total subdigital area (Table 6) and its contribution to the total subdigital area of the forelimbs, hindlimbs, and combined subdigital area is strikingly consistent between animals (Table 6). Although the pedal OB distal area is absolutely larger than that of the manus, each contributes to the total subdigital area of the forelimbs and hindlimbs in essentially equal proportions, ranging from 43.9% to 52.8% of manual and 44.1% to 52.1% of pedal subdigital area (Table 6). Some of the enlarged scales of the intermediate region (Figure 12a,b—as illustrated for digit IV) are seta‐bearing, so the distal region alone (Rothier et al., 2022) is not sufficient to demarcate the proximal boundary of the toe pads and underestimates toe pad area. Furthermore, the OB approach is not applicable in an identical manner to all digits because of differences in phalangeal number. For digits with an intermediate region, toe pad area is essentially equivalent to that accounted for by the LDT zone of the tendinously based (TB) approach for some digits and is greater than the latter for others (Figure 8). For digits that lack an OB intermediate zone, the area of the distal region is always smaller than that of the TB LDT region (Figure 8). The TB approach is unaffected by such variability and is equally applicable to all digits.

When considering only two anatomical regions throughout the digits (Figure 2), the TB LDT region is the major contributor to subdigital area of the manus and pes, accounting for 76.5% to 86.4% of manual and 81.4% to 86.7% of the pedal total (Table 7). The consistency of a large TB LDT area across the digits (Table 7) suggests the importance of scale contact area within this region for proper functioning of the adhesive system. All scales of the TB LDT region bear epidermal filaments arrayed in tetrads with spatulate tips (true setae).

The more distal sectors of the digits are actively hyperextended during digital release (Figure 14f). It has been hypothesized that the LDT system (Figure 3d) and the elaboration of a complex blood sinus system (Figure 3c) are linked to the effective deployment of the subdigital setae (Russell, 1981a, 1986, 2002). This integrated system is involved with the application of both the perpendicular and parallel preloads (Autumn et al., 2000) that are necessary for maximizing the frictional adhesive forces (Autumn et al., 2006) involved in seta‐based attachment in geckos. The structural continuity between the LDTs and the setae via the intervening highly modified dermis of the scales (Russell, 1976) relates directly to the application of a controlled parallel loading regime of the setae (see below). This, in combination with the expression of vascular reticular networks (Figure 3c) in these modified scales (Russell, 1981b) and the restriction of true setae (with spatulate tips—Garner & Russell, 2021) to the TD LDT region of the digits (Figure 12a,b), allows anatomical and functional definition and delimitation of the toe pads of *Gekko gecko*.

The mechanical relationship of the LDTs to the setae of the definitive toe pad scales (Imburgia et al., 2019) underpins the metaphorical titular recognition of “Geckos as springs” by Gilman et al. (2015), who demonstrated that adhesive system compliance in geckos is directly related to the maximum adhesive force able to be generated by the adhesive system. The LDTs behave as mechanical springs of finite stiffness, providing controllable resistance in the direction of loading. Recognition of this relationship led to the development of the highly effective synthetic adhesive material Geckskin® (Savage, 2015). The vascular sinus and reticular network complex (Russell, 1981b) underlying the stiffened integument of the ventral surface of the scales of the toe pad proper (Figures 3c and 4a–e) provides the “soft core” that enhances conformability of the “stiff skin” with the substratum (Glasser & Langowski, 2023; Russell, 1981b, 2016).

Enlarged scales located more proximally on the digits constitute folds that are relatively flat (Figure 4a, scales 2, 3, and 4). These are lamellae (Peters, 1964—“any one of a group of soft, overlapping plates, lying in ranked series…most often used herpetologically for the transverse plates on the underside of digits in many lizards”) that imbricate much less than the tendon‐bound, more distal scansors (Figure 4a). Almost the entirety of their ventral surface is exposed to the locomotor substratum (Figure 4g). Lamellae do not house components of the reticular network (Figure 3c) associated with the digital vascular sinus system (Russell, 1981a), nor are they served by branches of the LDT system (Figure 3d). Thus, their epidermis is not tightly bound to underlying tissues (Russell, 1986). They do, however, bear relatively long, branched prongs (Figures 4g, 11, and 12) that, like the friction‐promoting integumentary filaments of chameleon digits (Khannoon et al., 2014; Spinner et al., 2014), probably enhance traction with the substratum. *Gekko gecko*, like the phyllodactylid gekkotan *Tarentola mauritanica* (Linnaeus, 1758) (Russell & Higham, 2009), holds its toe pads proper (as defined in this contribution) in a permanently hyperextended configuration (Figure 14f) when running on horizontal substrata (Russell, 2002), contacting the surface solely with the more proximally situated lamellae. The boundary between the prong‐bearing lamellae and the seta‐bearing scansors segregates the digits into two functional regions that interact with the substratum in different ways. The distinction between these regions is apparent when viewing the digits of *G. gecko* in their hyperextended state (Figure 14f): only those enlarged subdigital scales that are intimately associated with the LDTs are held clear of the substratum, the enlarged scales underlying the basal sectors of the digits remaining in contact with the locomotor surface.

The morphological distinction between those scales associated with the LDTs and those proximal to them reflects functional differences (Russell, 1981a, 1986, 2002). Subdivision of the digits into the TB LDT and basal functional regions allows for more meaningful comparisons to be made between the same zones from digit to digit (sets of anatomically identical anatomically configured scales), as well as between species, despite variation in the number of functionally equivalent scales between digits both within and between species (Bergmann & Russell, 2003).

Assessment of toe pad area on the basis of some aspect of relative scale size alone, or on the basis of the association of scales with only the penultimate phalanges (when phalangeal number varies between digits—see Figure 2) has led to inconsistent results concerning the scaling relationship between toe pad area and body size, likely because scales not forming part of the anatomically restricted, seta‐bearing integrated complex have either been incorporated or excluded. Scaling relationships between body size and pad area have focused either on predetermined parameters (Bauer & Good, 1986) or undisclosed boundaries (Irschick et al., 1996) of the toe pad when assessing toe pad area. Bauer and Good (1986) predicted that toe pad area of *Gekko gecko* would scale in proportion to mass or body length, but found no such relationship. Their assessment of toe pad area did not scale in proportion to the weight it supports, leading them to state that toe pad area is only an estimate of true contact area that is actually determined by characteristics of the setae. Irschick et al. (1996) compared the clinging ability of a variety of pad‐bearing geckos, skinks, and anoles. A tight correlation between pad area and clinging ability was observed, but pad area did not scale with body size. Removing the effects of body size left approximately 50% of the variation in clinging ability unexplained (55% across multiple species of *Anolis*; Elstrott & Irschick, 2004), suggesting that factors other than size are involved (Garner et al., 2019, 2022; Gillies et al., 2014; Peattie & Full, 2007). These noted discrepancies may, however, also be the result of comparison of subdigital areas not reflective of the actual toe pads within and between species. Different methods of determining the limits of the functional toe pads will affect the assessment of area.

The enlarged scales of digit III of *Gekko gecko* included in the TB LDT region of the digit, constituting what we define as the toe pad proper, are colored purple in Figure 14a. Six enlarged scales occur proximal to that region. Five of those would be incorporated into the measurement of toe pad area applying the criterion of Peterson and Williams (1981) and all six when applying the criterion of Bauer and Good (1986) (Figure 1b). Other approaches, where there is no statement about which scales are included in the putative toe pad, would incorporate most, if not all, of these. This more proximal zone of enlarged scales increases the assessment of toe pad area considerably, with all six of these scales accounting for approximately an additional 37% of the area accounted for by the toe pad proper (Figure 14a, scales indicated in purple). Conversely, the toe pad proper accounts for only about 73% of the area obtained by incorporating all enlarged subdigital scales. Contrastingly, only the distalmost 10 scales colored purple in Figure 14a would be included in the assessment of the area of the toe pad as defined according to the OB criterion of Rothier et al. (2022), resulting in an underestimation of toe pad area by about 30%.

Although it requires a greater level of investigation at the outset, we encourage those interested in comparative studies of toe pad area, both within and between species, to firstly establish what scales on the digit(s) of interest are justifiably assigned, on anatomical grounds, to toe pad membership before further measurement and analysis are undertaken. Different lineages may well show different patterns of relationship and different structural features because of differences in digit form (Garner & Russell, 2026; Russell, 1976; Russell & Gamble, 2019), ecological relationships (Griffing, Gamble, et al., 2024), and substratum preferences. Such aspects can only be effectively addressed if these deeper patterns are appreciated. By confining estimates of toe pad area to the TB LDT components of the digits (i.e., those scales having direct and integrated association with the LDTs—see Russell, 1976), it should be possible to more effectively conduct cross‐species comparisons otherwise rendered difficult because of the varying form and structure of the subdigital region both within (Griffing, Gamble, et al., 2024) and between taxa (Garner & Russell, 2026, figures 2 and 4). Our results suggest that using the TB LDT region for assessing toepad area will provide a more consistent and comparatively appropriate assessment of toepad area.

Correct delimitation of gecko toe pads has far‐reaching implications, as measurements of toe pad area are commonly used as variables or covariates in both comparative and biomechanical studies of gecko toe pad form and function. Adhesive performance of gecko toe pads, for example, is often reported as adhesive stress (force per unit area) to account for variation in toe pad area between species and/or individuals. Previous work, therefore, likely underestimates adhesive stress of gecko toe pads because of overestimation of toe pad area.

The most direct approach to the identification of the LDT region would involve destructive sampling of the digits through stripping of the integument to determine which scales remain tightly bound to internal components (the LDTs). This approach may be prohibitive for studies focusing on endangered or protected species with strict permitting requirements, species of limited quantity in museum collections, or living individuals. We thus advocate that noninvasive methods for identification of the LDT region in live animals and/or nondestructive sampling methods for preserved specimens. Contrast‐based analysis of toe pad photographs, such as that employed by Pillai et al. (2020b), is one potential solution for live animal work, although additional study is needed to verify that the regions identified by this methodology correspond with the anatomical markers identified here. It is also possible that future contrast‐enhanced computed tomographic protocols may be developed to assist with the identification of tendinous structures. Currently, staining protocols for identification of soft tissues via diffusible iodine‐based contrast‐enhanced computed tomography (diceCT; Gignac et al., 2016) are not very effective for visualizing tendons.

Fortuitously, the findings of the current study can be combined with those of other lines of investigation to enable effective identification of the LDT region. In recent years, investigation of the embryonic development of gecko toe (and tail) pads has been undertaken (Alturk & Khannoon, 2020; Griffing et al., 2021, 2022; Griffing, Gamble, et al., 2024; Khannoon, 2015; van der Vos et al., 2018). Collectively, the results of these studies reveal that hypertrophy of the subdigital region occurs during development of the digits in the region in which the toe pad will be located (for taxa with both basal and terminal, leaf‐like pads; Griffing et al., 2022). This hypertrophied zone expands mediolaterally, swells and becomes divided into transverse ridges that will ultimately form the scansors. Such studies clearly demonstrate a heterochronic shift whereby the ancestral pattern of simultaneous expression of subdigital scales along the length of the digit is disrupted, with the presumptive scansors being expressed before scales located proximal and distal to them. These developmental findings (Alturk & Khannoon, 2020; Griffing et al., 2021, 2022; Griffing, Gamble, et al., 2024; Khannoon, 2015; van der Vos et al., 2018) do not, however, indicate the demarcation point between the adhesively effective scansors and the more proximal friction plates (Russell et al., 2015; Russell & Bauer, 1990a). The hypertrophy of the developing scansors, as seen externally (Griffing et al., 2022; Griffing, Gamble, et al., 2024) is associated with the development of deeper tissues that demarcate the toe pad scales proper from other scales of the digits. What can be deduced from figures 3 and 4 of Griffing et al. (2022) is the association of the developing toe pads with particular (but variable between taxa) sets of phalanges. The integrated nature of phalangeal and toe pad association is evident in gekkotan taxa in which toe pads are either reduced in size or effectively lost (Haacke, 1976; Higham et al., 2015; Johnson et al., 2005; Powell et al., 2017; Russell, 1976; Russell & Bauer, 2008), providing some guidance about how to associate the recently published developmental findings (Griffing et al., 2022) with other aspects of digit anatomy. Employing the images of digit IV of *Anolis* and the gecko taxa investigated by Griffing et al. (2022), we have developed a schematic representation (Figure 15) that correlates the proximal extent of the toe pads with particular interphalangeal joints (discernible in Griffing et al.'s [2022] figures). For basal toe pads (of both *Anolis* and the gekkotans *Correlophus*, *Lepidodactylus*, and *Hemidactylus*), the proximal margin of the toe pad proper lies at the boundary between the basalmost (first) and second phalanx, accords well with our more extensive morphological findings for *Gekko gecko* (see above). Interphalangeal articulations are locatable by radiography or specimen manipulation, this obviating the need for destructive sampling. Our findings for *G. gecko* indicate that the proximal extent of the adhesively competent toe pads occurs at the articulation between the basalmost and next most distal phalanx, regardless of the total number of phalanges in a given digit (Figure 16), and that this coincides with the proximal extent of the LDT region.

**FIGURE 15:**
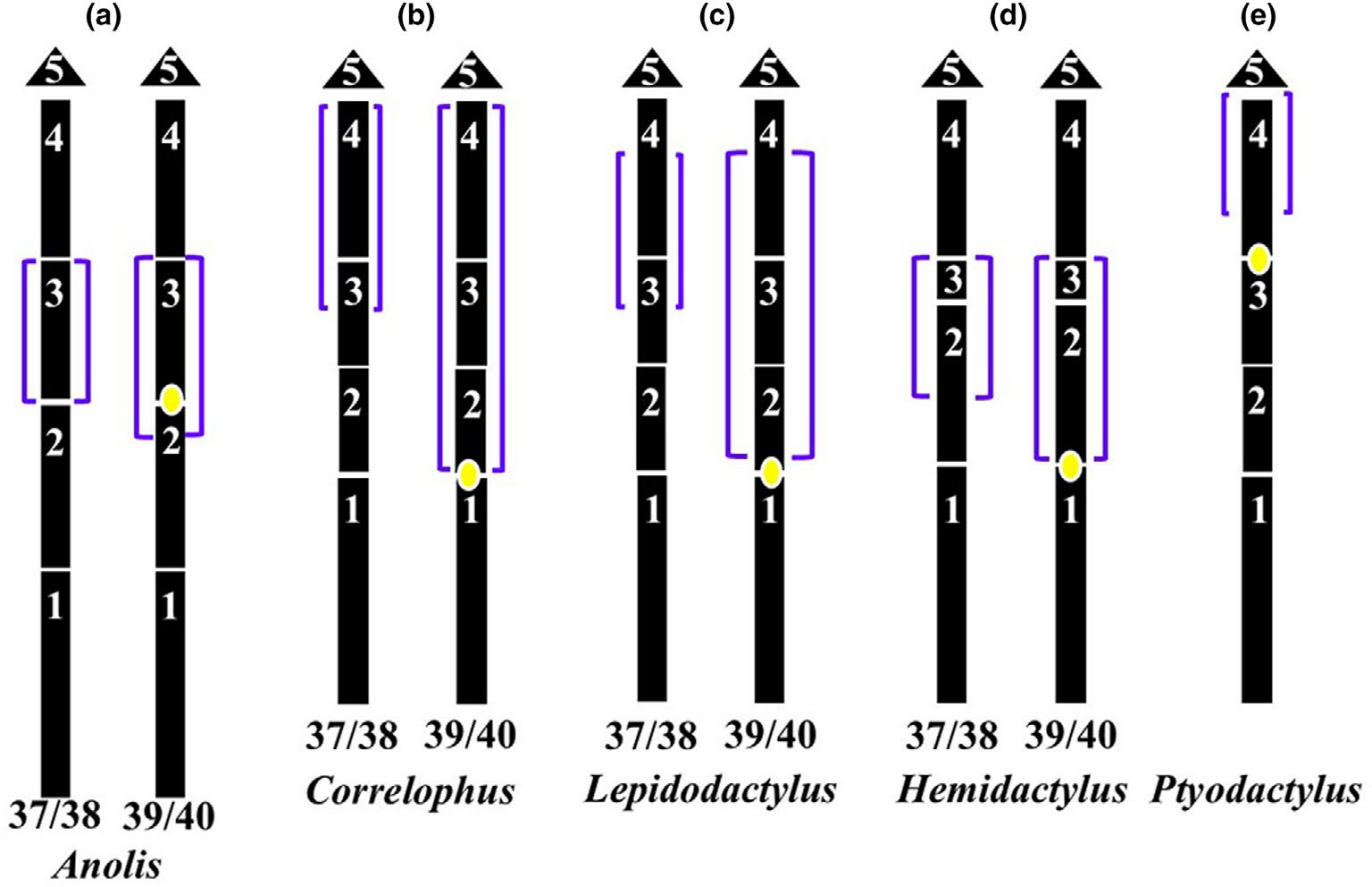
Schematic representation of toe pad development in relation to phalangeal associations in (a) *Anolis carolinensis*, and the geckos (b) *Correlophus ciliatus*, (c) *Lepidodactylus lugubris*, (d) *Hemidactylus turcicus*, and (e) *Ptyodactylus guttatus*. The location of the extent of the developing toe pads relative to the phalanges of pedal digit IV is indicated by purple bracketing lines at developmental stages 37/38 (first signs of the expression of presumptive scansors) and 39/40 (expression of the full proximodistal extent of the scansors) for a, b, c, and d. These associations are gleaned from figures 3 and 5b of Griffing et al. (2022), in which pertinent interphalangeal joints are evident, and skeletal anatomical data in Russell (1972, 1976), Russell and Bels (2001), and Russell and Garner (2023). Arabic numbers indicate phalanges (as in Figure 2) and yellow ovals with white outlines in stage 39/40 depictions (and for *Ptyodactylus* for which no staging data are available) indicate the location of the interphalangeal joint that marks the proximalmost extent of the toe pad and the LDT region. Differing phalangeal proportions for different species are approximated by the relative lengths of the numbered boxes.

**FIGURE 16:**
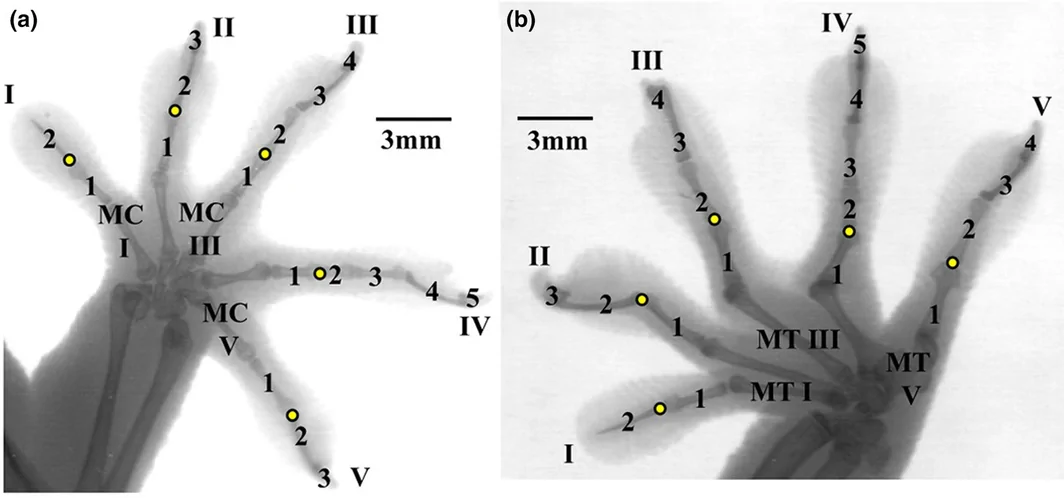
Radiographic dorsal views of the right manus (a) and right pes (b) of *Gekko gecko* (as in Figure 2, with identical labeling of digits and skeletal elements). Yellow circles with black borders indicate the interphalangeal joint in each digit that corresponds with the proximalmost extent of the adhesively effective toe pads (as identified in this study) and the LDT region of each digit.

Prior studies of the relationship between the LDTs of geckos and phalanges, for both basal and terminal, leaf‐like toe pads, can now be integrated with the findings of the current study. It is evident that the LDT region demarcates the toe pads and that this is associated with the presence of adhesively effective setae. Schematic representations of LDT/phalangeal associations in geckos (and anoles) (Russell, 1976; Russell & Bauer, 1989, 1990b) can be used to assist with the determination of pertinent interphalangeal articulations and thus the delimitation of the extent of the toe pads. There will likely be some difficulty in determining the toe pad boundary in taxa in which distal, leaf‐like toe pads encroach more proximally along the digits (Kukla et al., 2026; Russell & Gamble, 2019), but schematic representations of such cases (Russell, 1976, figures 3d,e and 4b; Russell & Bauer, 1989, figure 3; Russell & Bauer, 1990b, figures 7–10) can assist.

Our findings reported in this contribution indicate that identifying the LDT region of toe pads via the TB approach remains the only empirically supported method for delimiting the toe pad proper (that part of the subdigital surface bearing adhesively competent setae) of *Gekko gecko*. As such, we encourage future studies to adopt this approach by adopting the guidelines for gecko toe pad delimitation outlined above, so that a standardized metric, built upon clear anatomical and functional foundations, can be used to ensure accuracy of the measurement and facilitation of effective comparison between species and studies.

In addition to the broader implications of our study for delimiting the toe pads of gekkotan and anoline lizards, our results also demonstrate that toe pad area (i.e., LDT region area) varies from digit to digit within an autopodium (Figures 8 and 10; Tables 3 and 7) and between the manus and pes (Figures 8 and 10; Tables 3 and 7). Thus, simple extrapolation from the measurement of the toe pad area of a single digit, or single manus or pes, may not accurately reflect the totality of adhesively effective area, or potential clinging capabilities. Such relationships may vary from species to species, so they will need to be carefully considered in comparative studies. In *Gekko gecko*, pedal digits have longer phalanges (Figure 2) and carry more toe pad surface area than manual ones (Figures 8, 9, 10, Tables 2, 3, 6, 7, 8, 9). This is also the case in other species for which comparisons have been made (Russell & Oetelaar, 2016; Webster et al., 2009). Geckos may rely more on pedal digits in certain circumstances, such as when resting head down on a vertical surface (Russell & Oetelaar, 2016) or when descending inclined substrata (Birn‐Jeffery & Higham, 2014, 2016) by rotating their hind feet into a similar orientation to that adopted when resting head down (Russell & Oetelaar, 2016) and employing them as brakes. Manual digits cannot be reoriented in this way, perhaps in part accounting for proportionally larger hindlimb toepad area.

Our approach to clarifying the functionally defined boundaries of the toe pads of *Gekko gecko* also enables us to assess previous statements about the total toe pad area for all 20 digits as well as the structure, distribution, and dimensions of the subdigital filamentous fields. Various estimates have been published for important parameters of the adhesive system of *G. gecko*, but no comprehensive overview has been assembled.

The prior assumption that all setae are of equivalent configuration and distribution throughout the toe pads (as is inherent in statements about gecko toe pads in general—e.g., Autumn et al., 2000, 2002; Hill et al., 2011; Huber et al., 2007; Huber, Gorb, et al., 2005; Huber, Mantz, et al., 2005; Sun et al., 2005; Xu et al., 2015, 2018) implies that the scales with the greatest area will contribute more potential adhesive capacity than scales with lesser area (Tables 8 and 9). Although our estimates of setal tip patterns must be considered approximate at present, it is evident that setal tip dimensions and density change along the length of the toe pad (Figure 12a–d), indicating that there may be some level of local adjustment resulting in tip area varying much less than scale area along the majority of the TB LDT region of the digits. Total tip area per scale (Tables 8 and 9) is more constant along the length of the toe pad (except for the relatively small distalmost scales) than would be expected if extrapolation was done based upon scale area and uniform setal dimensions alone. Given that contact with the substratum at any footfall is likely to be patchy (Johnson et al., 2009; Johnson & Russell, 2009; Russell & Johnson, 2007a, 2007b, 2014), potentially equitable adhesive capacity from any scale, or part thereof, of the toe pad might be expected. Permutation of setal tip area per unit area of the toe pad to achieve equitability is suggested by the data presented in Tables 8 and 9. Changing setal dimensions, branching patterns and numbers of setal tips per unit area along the length of the toe pad (and, more subtly within the confines of each scale—Russell & Garner, 2021) are suggestive of regionalized permutation that fosters equitability of adhesive capacity for all regions of the toe pad even though the dimensions of the filaments change (due to the functional properties of the geometrically integrated setal fields [Johnson & Russell, 2009; Russell & Garner, 2021]).

Our data also allow us to consider the potential magnitude of the entire digital adhesive apparatus of *Gekko gecko*. The data presented in Tables 3, 8, and 9 provide the foundation for extrapolating about the properties of the toe pads within and between digits. In Table 10, we present data relating to the estimation of the contributions of each digit of the manus and pes to the total area of the TB LDT regions of the digits, these regions comprising the mechanically controllable toe pads (Figure 3).

**TABLE 10:** Extrapolated values for the total number of setal stalks and setal tips on all 20 digits of the tendon‐based lateral digital tendon (LDT)‐delimited toe pads of *Gekko gecko*.

	Percent of total LDT area for autopodium (data from Table 3)	Total number of setal stalks (data from Tables 8 and 9)	Number of setal tips calculated for means of manual scales 19, 15, 12, 11, 8, and pedal scales 18, 15, 12, multiplied by 13 (data from Tables 8 and 9)
Digit manus			
I	16.4		
II	18.4		
III	23.5		
IV	21.8	262,112	116,764,950
V	19.9		
Calculated total for total single manus based upon percentage representation		1,202,349	401,428,900
Digit pes			
I	15.9		
II	17.2		
III	22.7		
IV	21.3	353,672	163,270,029
V	22.65		
Calculated total for total single pes based upon percentage representation of digits		1,660,432	766,525,958
Calculated total for left and right manūs and pedes		5,725,562	2,335,909,716

Regarding total toe pad area of *Gekko gecko*, previous estimates for all 20 digits range from 227 mm^2^ (Sun et al., 2005) to 400 mm^2^ (Autumn et al., 2000). Our observations yielded a mean value of 402 mm^2^, with 168 mm^2^ contributed by the manūs and 234 mm^2^ by the pedes.

Previous estimates of the total number of setae per digit are rather nebulous, being stated to be “hundreds of thousands” (Autumn et al., 2006) to “millions” (Xu et al., 2018). Our estimates of approximately 262,000 setal stalks for digit IV of the manus and 353,000 for digit IV of the pes (Table 10) accord with the statement of Autumn et al. (2006) but fall far short of the estimate of “millions.” We estimate that there are about 1,200,000 setae carried by each manus and about 1,660,000 by each pes (Table 10). For all 20 digits, this provides an estimate of over 5,725,000 setae for all four autopodia (Table 10), a large number that would fall somewhere within the broad extrapolated range obtainable from the estimate of Autumn et al. (2006), but far below the 20 million or more that can be extrapolated from Xu et al.'s (2018) statement. An extremal statement by Guo et al. (2007) that each enlarged scale (their “valvulae”) of the toe pads of *Gekko gecko* bears millions of setae would yield an astronomically large number for each digit, let alone all four autopodia.

Prior reports of setal lengths for *Gekko gecko* fall within the range of 30–130 μm (Autumn et al., 2000; Huber, Gorb, et al., 2005). Our observations for the TB LDT regions of the digits (Figure 11a,b) indicate a maximum length in the region of 130 μm and a minimum of about 50 μm. More proximally (Figure 11a,b), filaments span from about 60 μm down to those <10 μm in length, but none of these bear spatulate tips (Figure 12a,b) and so do not qualify as setae (Garner & Russell, 2021).

Statements about the basal diameter of setal stalks span a range from 2 to 10 μm for *Gekko gecko* (Huber et al., 2007; Huber, Gorb, et al., 2005). For the TB LDT region of the digits, we found the setae to have basal diameters between 3.5 and 4.5 μm (Figure 11c,d). Further proximally, this diminishes to as little as 1.0 μm. In no instances did we observe diameters approaching 10 μm.

Estimates of setal density for *Gekko gecko* range from 5000 (Autumn et al., 2000) through 5300 (Sun et al., 2005) to 14,400 per mm^2^ (Autumn et al., 2002). For the TB LDT region of the digits, we recorded densities ranging from about 16,000 to 24,000 setal stalks per mm^2^, with the density of stalks increasing at the proximal end of the toe pad (Tables 8 and 9). Stalk densities proximal to the TB LDT region of the digits increase considerably from that encountered in the TB LTD region (Tables 8 and 9), but these filaments are not setae (Figure 12a,b). Densities in the region of 5000 setae per mm^2^ (Autumn et al., 2000; Sun et al., 2005) were never encountered in our observations, but our findings accord quite closely with the 14,400 per mm^2^ of Autumn et al. (2002) for most of the extent of the toe pads.

Our observations (Tables 8 and 9) indicate that setae carry in the region of 250–560 spatulate tips, with the number of tips per seta increasing from proximal to distal along the length of the TB LDT region of the digit. We note, however, that isolating individual setae to count the tips exclusive to them is challenging, especially when trying to survey entire setal fields. Our estimates are thus based upon a marriage of setal density data gained from frontal sections of the setal fields (Figures 5 and 14) and tip counts obtained by examining SEM images of setal fields in planar view (Figure 4). More nuanced approaches may be needed to clarify this issue further.

The number of spatulate tips per seta reported in the literature covers a broad range, with Autumn et al. (2000), Huber, Gorb, et al. (2005), and Huber, Mantz, et al. (2005) indicating that individual setae carry from 100 to 1000. Autumn et al. (2002), Sun et al. (2005), and Xu et al. (2018) state that individual setae end in hundreds of tips, while Huber et al. (2007) indicate that setae may carry hundreds to thousands of spatulae. Our observations (Tables 8 and 9) indicate that setae exhibit in the region of 250–560 spatulate tips. Estimates of tip numbers in the hundreds by previous authors accord with our observations, but we did not encounter the 1000 or more mentioned by some (Autumn et al., 2000; Huber et al., 2007; Huber, Gorb, et al., 2005; Huber, Mantz, et al., 2005).

Previous assessments of the dimensions of the spatulae carried on the setae of *Gekko gecko* have implicated an array of sizes. The statement by Autumn et al. (2000) that they range in width from 0.2 to 0.5 μm incorporates the 0.23–0.44 μm range advocated by Autumn et al. (2002), and the 0.2 μm width advanced by Huber, Gorb, et al. (2005) and Huber et al. (2007). Sun et al. (2005) indicated the tips of the setae are 0.1 μm wide. Our measurements of setal tip widths span from 0.1 to 0.26 μm (Tables 8 and 9), in general accordance with those of previous investigators, although we note that the variation we report follows a pattern along the length of the toe pad (Tables 8 and 9) that is integrated with other sequential proximodistal changes in dimensions of the setae (Figures 11 and 12) and configuration of the setal fields (Figure 13). Statement of a single “exemplar” dimension for such attributes of setae as representative of the entire toe pad is too simplistic.

Employing the estimates for setal tips on scales 19, 15, 12, 11, and 8 of the manus (Table 8) and scales 18, 15, and 12 of the pes (Table 9), we calculated a mean for each of these and multiplied this by 13 (the number of scales incorporated in the TB LDT region of digit IV) to arrive at an estimated total for these digits (Table 10): 116,764,950 for the manus and 163,270,029 for the pes. Extrapolating this estimate to include all 20 digits, based upon their proportional contribution to the total area of the TB LDT toe pads, yields in excess of 2.3 billion spatulate tips for the entire system (Table 10). Autumn et al. (2000), Huber, Gorb, et al. (2005), and Huber, Mantz, et al. (2005) posited that the total number of spatulate tips carried by the setae on all 20 digits of *Gekko gecko* was about 1 billion. Our estimation of in excess of 2.3 billion spatulate tips for the entire system (Table 10) is more than double that previously suggested. Placing this into what we feel is readily appreciable perspective, this estimate of 2.3 billion spatulate tips is over 10 times greater than the total number of letters and numbers in the gargantuan 10‐volume first edition of the Oxford English Dictionary (Winchester, 2003) that was finalized in 1928 and took 71 years to compile.

## 5 CONCLUSIONS

We collected comprehensive morphometric data of the subdigital scales and associated epidermal filaments of Tokay geckos (*Gekko gecko*) to attempt to objectively define what constitutes the “adhesive toe pad.” We introduced and compared two different anatomically grounded approaches (OB and tendinously based [TB]) that define subdigital regions based upon integration with internal structures rather than being based purely on external features. Relative to OB and TB determination of toe pad delimitation, previously employed alternative, scale‐based delimitation schemes (Bauer & Good, 1986; Beuttell & Losos, 1999; Peterson & Williams, 1981) universally overestimate functional toe pad area. The anatomically based OB and TB approaches to toe pad delimitation are not equivalent in their outcome; proximal and intermediate regions of the toe pad identified in the OB approach include some scales that do not bear epidermal filaments considered true setae (Garner & Russell, 2021). The LDT region of the TB approach, however, ubiquitously and exclusively incorporates scales whose surfaces only bear true setae. Considering that true setae are the only filaments empirically demonstrated to facilitate whole‐animal adhesion to vertical, low‐friction substrata (Garner & Russell, 2021), we contend that the toe pad proper should be defined as the LDT region as described here. We provide information about how to approximate the LDT region of gecko digits in general without having to destructively sample specimens or sacrifice living animals.

## AUTHOR CONTRIBUTIONS

A.P.R. designed the project. S.M.D. acquired the data. S.M.D., A.P.R., and A.M.G. analyzed and interpreted the data. A.P.R. wrote early drafts of the manuscript. A.M.G. and S.M.D. provided critical reviews of earlier drafts. A.P.R., S.M.D., and A.M.G. collectively finalized the article and approved it for submission.

## Data Availability

The data that support the findings of this study are available from the corresponding author upon reasonable request.
